# Supplementary material for: Universal peptide synthesis via solid-phase methods fused with chemputation
Source: Nat Commun. 2025 Aug 8;16:7322. doi: 10.1038/s41467-025-62344-2 (PMC12334676; doi:10.1038/s41467-025-62344-2)
Supplement: Supplementary file 1 — Supplementary Information [file 41467_2025_62344_MOESM1_ESM.pdf]

Supplementary Information

Universal Peptide Synthesis via Solid-Phase Methods fused with Chemputation

Jacopo Zero[1†], Tristan J. Tyler [1†], Leroy Cronin[1*]

1 - School of Chemistry, University of Glasgow, University Avenue, Glasgow G12 8QQ, UK.

† Equal contribution

Email: lee.cronin@glasgow.ac.uk

Contents

1. Supplementary Tables 3
2. Materials and methods 5
3. χDL and Chemputer framework 6
   3.1 Software stack 6
   3.2 Execution of χDL files for automated synthesis 6
   3.3 Chemputer hardware 7
   3.3.1 SPPS Chemputer full assembly 8
   3.3.2 Frame and shelving 10
   3.3.3 SPPS reactor custom glassware 13
4. χDL blueprints 14
5. Fully automated synthesis of peptides 28
   5.1 ACP(65-74) (1) 28
   5.2 18A (2) 29
   5.3 GHRH(1-29) (3) 30
   5.4 Semaglutide – on-resin sidechain functionalization (4) 32
   5.5 NYAD-13 – on-resin ring-closing metathesis (5) 34
   5.6 Peptide–bismuth bicyclic complex (6) 36
   5.7 CuAAC “click” 6-FAM fluorescent labelling of penetratin (7) 38
   5.8 Late-stage diversification via cysteine arylation stapling (8-11) 40
   5.9 Capitellacin – directed oxidative folding (12) 45
   5.10 OPA-mediated sidechain cyclization and maleimide derivatization (13) 48
   5.11 α-amanitin analogue synthesis via native chemical ligation (14) 50
   5.12 N-Methyl-18A (15) 52
6. Supplementary References 54

1. Supplementary Tables

Supplementary Table 1: Condition optimization for the fully automated synthesis of the three peptide sequences ACP(65-74) (**1**), 18A (**2**), and GHRH(1-29) (**3**) on the Chemputer.

Peptide Sequence	#	Coupling reagent	Coupling time (min)	Cleavage time (h)	Crude purity (%)	Crude yield (mg / %)	Synthesis time (hh:mm)
ACP(65-74)	**1**	HATU	30	2	90	53 / 41	20:49
		HBTU	30	2	87	48 / 36	20:52
		PyBOP	30	2	89	67 / 51	20:51
		HATU	15	2	85	44 / 31	18:39
		HATU	60	2	87	58 / 43	25:20
		HATU	30	4	90	94 / 72	22:53
18A	**2**	HATU	30	2	93	195 / 67	34:26
		HATU	30	4	94	217 / 76	36:25
GHRH(1-29)	**3**	HATU	30	2	87	261 / 59	49:54

Supplementary Table 2: Calculated and observed masses of synthesized peptides. Observed masses were measured via RP-HPLC ESI-MS.

#	Chemical formula [M]	Calculated mass (Da) $[M+H]^+$	Observed mass (Da) $[M+H]^+$
**1**	$C_{47}H_{74}N_{12}O_{16}$	1063.5	1063.5
**2**	$C_{108}H_{160}N_{24}O_{28}$	2242.2	2242.2
**3**	$C_{147}H_{241}N_{41}O_{42}$	3253.8	3253.9
**4**	$C_{187}H_{291}N_{45}O_{59}$	4112.1	4112.2
**5**	$C_{87}H_{136}N_{18}O_{19}$	1738.0	1738.0
**6**	$C_{58}H_{82}BiN_{17}O_{16}S_3$	1578.5	1578.6
**7**	$C_{133}H_{190}N_{38}O_{27}S$	2784.4	2784.4
**8**	$C_{36}H_{44}F_4N_8O_{10}S_2$	889.3	889.3
**9**	$C_{42}H_{44}F_8N_8O_{10}S_2$	1037.3	1037.3
**10**	$C_{43}H_{46}F_8N_{10}O_{11}S_2$	1095.3	1095.3
**11**	$C_{48}H_{44}F_{13}N_8O_{10}PS_2$	1235.2	1235.3
**12a**	$C_{105}H_{173}N_{39}O_{26}S_4$	2525.2	2525.3
**12b**	$C_{105}H_{171}N_{39}O_{26}S_4$	2523.2	2523.3
**12c**	$C_{99}H_{159}N_{37}O_{24}S_4$	2379.1	2379.2
**13a**	$C_{50}H_{74}N_{14}O_{16}S$	1159.5	1159.6
**13b**	$C_{58}H_{76}N_{14}O_{16}S$	1257.5	1257.6
**13c**	$C_{82}H_{89}N_{15}O_{23}S$	1684.6	1684.6
**14a**	$C_{46}H_{66}N_{12}O_9S$	963.5	963.5
**14b**	$C_{54}H_{70}N_{10}O_{11}S_2$	1099.5	1099.5
**14c**	$C_{46}H_{62}N_{10}O_9S$	931.4	931.5
**15**	$C_{110}H_{164}N_{24}O_{28}$	2270.2	2270.2

2. Materials and methods

Solvents and reagents were obtained dried and distilled from commercial sources and used as purchased unless stated otherwise. H_2O was purified using an Elga Purelab Chorus H_2O-purification system.

Characterization via HPLC analysis was assessed on a Thermo Dionex Ultimate 3000 HPLC system equipped with an LPG-3400 RS pump, WPS-3000TRS autosampler, TCC-3000SD column compartment, and DAD3000 diode array detector. The HPLC was connected to a Bruker Maxis Impact II HDMS Q-TOF spectrometer (Bruker Daltonics). Samples were injected for chromatographic separation on an Agilent Poroshell 120 EC-C18 column (2.7 µm, 4.6 x 150 mm), eluting at 1 mL min^{-1} with mobile phase A being H_2O + 0.1% formic acid and mobile phase B MeCN + 0.1% formic acid, detecting UV (λ = 214, 220, 254, and 280 nm). The total run time was 73 minutes, with the LC method as follows: 0 min – 1% B, 60 min – 80% B, 62 min – 100% B, 66 min – 100% B, 68 min – 1% B, 73 min – 1% B. Alternatively, the total run time was 26 minutes, with the LC method as follows: 0 min – 0% B, 4 min – 10% B, 16 min – 70% B, 19 min – 100% B, 23 min – 0% B, 26 min – 0% B. Column compartment was set at 30 °C. MS measurements were taken in positive electron spray ionization mode (ESI+) with a mass range of m/z 50–2000 using the following parameters: capillary tip 4500 V, end plate offset -500 V, nebulizer 2.0 bar, dry gas 10.0 L min^{-1}, dry temperature 200 °C, quadrupole ion energy 5 eV, and collision energy 5 eV. Peptide purity was assessed by RP-HPLC at 214 nm using the BrukerAnalysis v4.1 software suite. Yields, both crude and isolated, were calculated based on resin loading, adjusting for TFA counterions at free N-termini and positively charged residues (Arg, Lys, and His), as well as purity values obtained from HPLC-MS. HPLC purifications were performed using an Agilent Technologies 1260 Infinity system equipped with an YMC-Actus Triart C18 column (100 x 30.0 mm, 5 µm, 12 nm), utilizing a linear gradient of 10-80% MeCN over 60 min at a flow rate of 20 mL min^{-1}. Fractions containing target product mass at the highest purity (>95%) were collected and lyophilized.

Laser Cutting was performed on a Monster1060 CO_2 Laser system (ML1060 130 W) from Radecal with the RDWorksV8 software. The applied parameters are summarized in the Supplementary Table 3 below.

Supplementary Table 3: Settings for laser cutting acrylic shelves.

Material	Laser power	Speed	Air	Laser through mode	Air pressure	Flow rate
6 mm acrylic	70% – 85%	10 mm/s	On	Enabled	0.3 MPa	33 L/min

3. χDL and Chemputer framework

3.1 Software stack

The software stack required to execute χDL files on a Chemputer platform has been installed and used as previously described without further modifications[1]. Further information is available from the corresponding author upon request.

3.2 Execution of χDL files for automated synthesis

Representative example of a full automated synthesis procedure on the Chemputer is identified by a χDL file, a graph file, and a Python execution script. The χDL file is written in the XML markup language and saved as a **.xdl** file. Writing and editing of such files is carried out in any preferred editor software (e.g. Visual Studio Code). The graph files are written in the json format and saved as **.json** files. These are created and edited via the ChemIDE web application graphical user interface (GUI; https://croningroup.gitlab.io/chemputer/xdlapp/). Finally, a short Python script is used to finalize the automated execution of χDL files on the Chemputer platform as outlined in the Supplementary Fig. 1 below:

Supplementary Fig. 1: General Python script used to execute χDL scripts on the Chemputer platform.

```
# Importing of the Python libraries needed to execute a XDL synthesis script
# on the Chemputer platform.
from xdl import XDL
import ChemputerAPI
from chempiler import Chempiler
from chemputerxdl import ChemputerPlatform

# Definition of the XDL and graph input files.
experiment_name = "SPPS"
graph_file = experiment_name + ".json"
xdl_file = experiment_name + ".xdl"

# Loading of XDL synthesis script.
# If working with many Blueprints, it is convenient to define a working
# directory where the Blueprint XDL files are stored.
x = XDL(
    xdl_file,
    platform=ChemputerPlatform,
    working_directory=r"C:\Users\User\Chemputer\Blueprints",
)
x.prepare_for_execution(graph_file)

# Connection of the physical hardware through the platform controller
# (Chempiler).
c = Chempiler(
    experiment_name,
    graph_file=graph_file,
    output_dir=experiment_name,
    simulation=False,
    device_modules=[ChemputerAPI],
)

# Execution of the XDL digital synthesis script using the Chempiler.
x.execute(c)
```

The above script can be saved as a **.py** file and directly executed. Alternatively, an interactive Python notebook (such as Jupyter) can be used to execute each step individually, allowing for more flexibility when setting up an automated chemical reaction. All syntheses presented below were executed using a Jupyter notebook.

3.3 Chemputer hardware

The Chemputer components utilized in this work, specifically: Chemputer pumps, Chemputer valves, pneumatic controller, jacketed filter (Precipitating unit), magnetic hotplate stirrer, and pH sensor, were built and used as previously described[1,2]. However, the general assembly and connectivity of such components was optimized for this work to reduce the overall footprint of the platform, by taking advantage of the modular nature of the Chemputer, and to aid the

automated execution of the carried out syntheses. Unique elements are described in the following sections.

3.3.1 SPPS Chemputer full assembly

The base modules required for the synthesis of peptides on solid support (Supplementary Fig. 1) included a simple backbone of three pumps and three valves. A further seven valves were then connected in a “daisy-chain” manner to the backbone valves. Five of these were used to accommodate reagents, while the remaining two are the auxiliary valves for the filter modules. Due to the flexible nature of the system, more valves can be readily introduced in the same manner as required by the synthetic procedure. Modules are connected via polytetrafluoroethylene (PTFE) tubing with 1.6 mm outer diameter and 1.0 mm inner diameter to minimize dead volumes during solution transfer. Exceptions included the tubing going to waste, feeding inert gas and vacuum to the filters, and connecting the filters to the backbone which have an outer diameter of 3.2 mm and an inner diameter of 1.5 mm. Pressurized nitrogen gas is connected from the pneumatic controller to the filters via the auxiliary valves. The solenoids on the pneumatic controller are then used to define the high active pressure (0.5 bar, 1 mL min^{-1}) used to sparge the resin for mixing, and low passive flow (0.5 bar, 0.5 mL min^{-1}) used to gently agitate the resin and reduce excessive solvent evaporation. Vacuum, provided via a diaphragm pump (500 mbar) is also connected to the filters via the auxiliary valves. The remaining hardware, unless stated otherwise, was used without further modifications, as extensively described in previous publications[1,3,4].

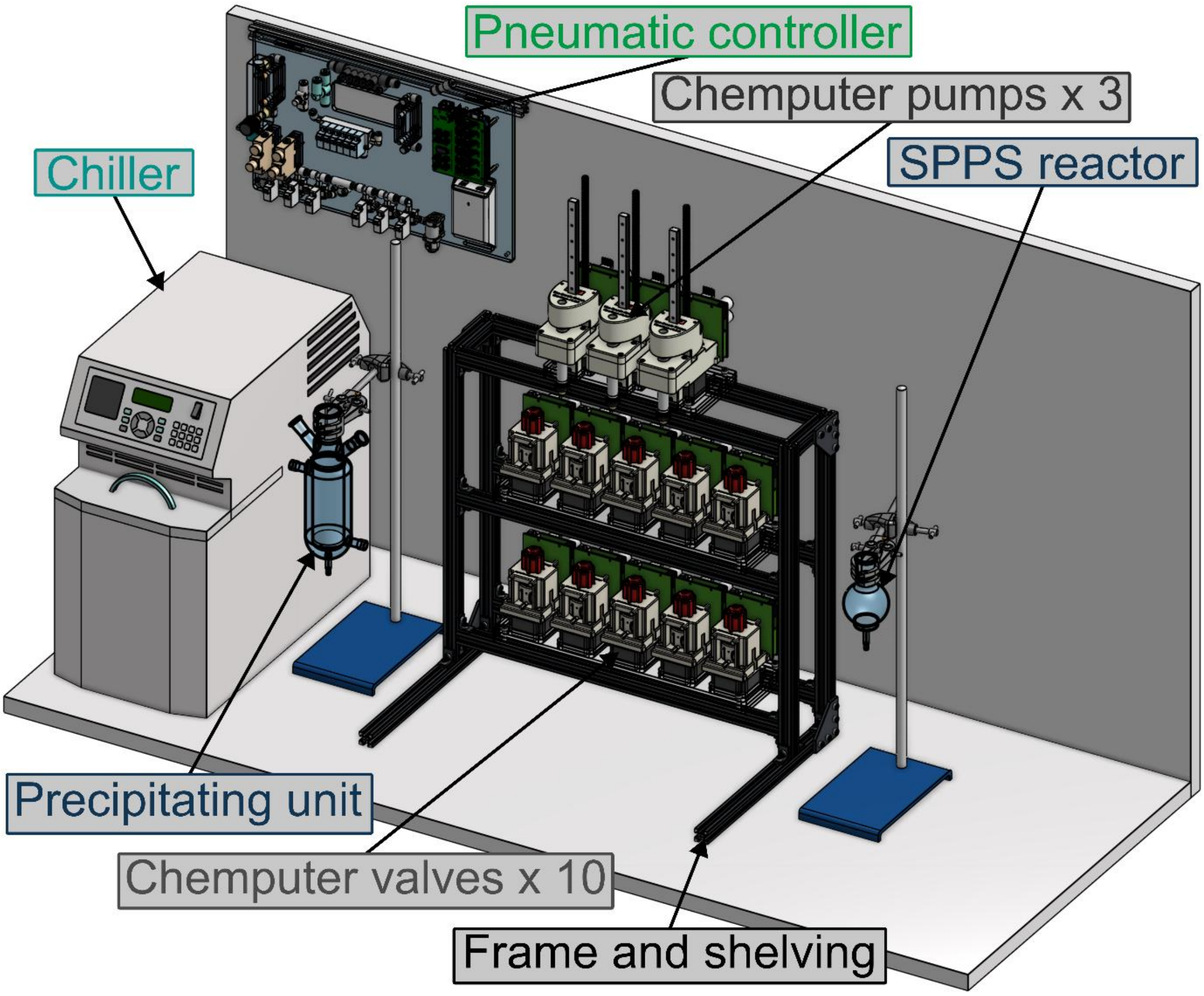

Supplementary Fig. 2: Model representation for the full SPPS Chemputer assembly depicting the base modules required for the fully automated solid-phase synthesis of peptides.

The connectivity between modules for the base setup of the SPPS Chemputer is represented in the digital graph (Supplementary Fig. 3).

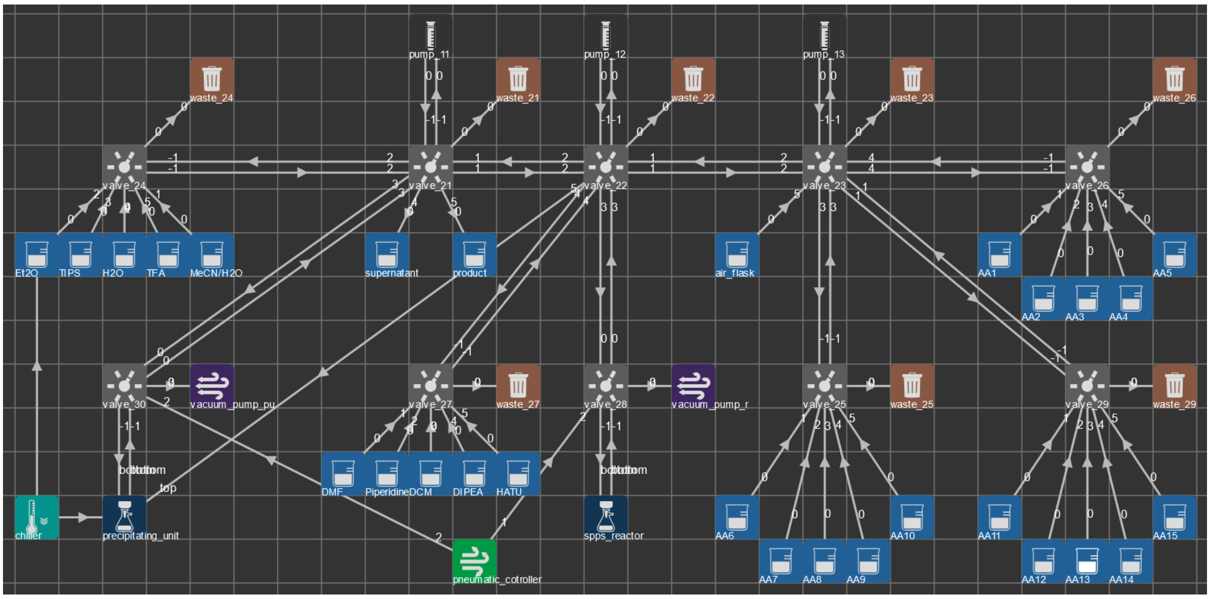

Supplementary Fig. 3: Generic digital graph representation of the connectivity between modules of the base setup for the SPPS Chemputer.

The base module can then be easily expanded with further valves or the necessary modules to carry out specific synthetic procedures as required. The layouts used throughout this project are represented by the digital graphs provided in the Supplementary Materials.

3.3.2 Frame and shelving

A unique frame and shelving setup was built to accommodate the Chemputer components utilized in this work as depicted in the Supplementary Fig. 4.

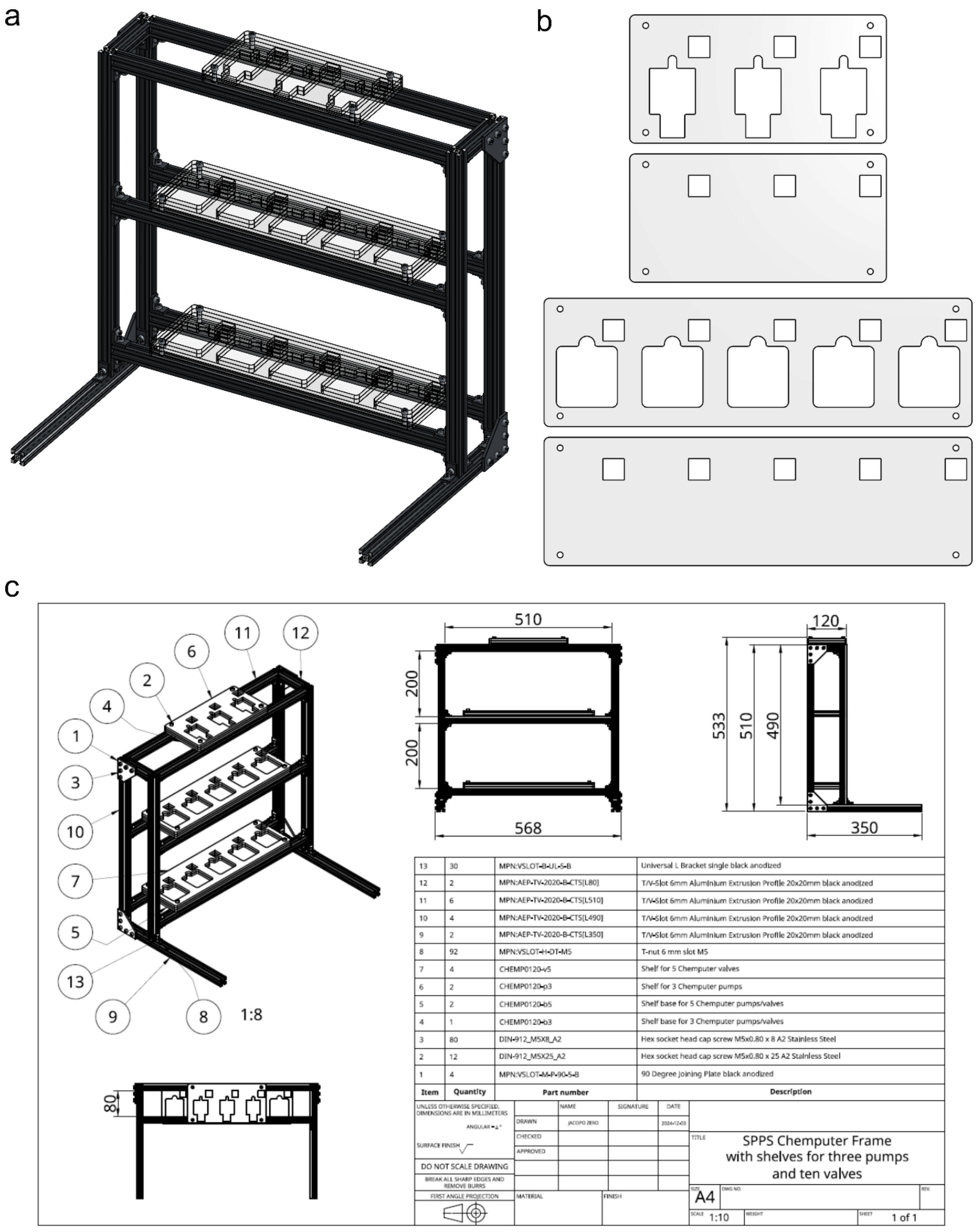

Supplementary Fig. 4: Frame and shelving setup for the SPPS Chemputer. **a**, Complete frame and shelving assembly for the SPPS Chemputer including three pumps and ten valves. **b**, 6 mm laser cut acrylic holders for three pumps and five valves. **c**, drawing with suggested dimensions for the frame and shelving assembly.

The frame was made using OpenBuilds® parts (Supplementary Table 4) where V-Slot rails of different lengths were joined with 90 Degree Joining Plates or L-Brackets using T-nuts and

M5 hex socket head cap screws. The shelves for the three pumps and the ten valves were laser cut on 6 mm acrylic panels and screwed onto the frame using T-nuts and M5 hex socket head cap screws. For Chemputer pumps, two three-pumps holders and one three-pumps shelf base were laser cut, while, for Chemputer valves two sets of five-valves shelves were laser cut. These included two five-valves holders and a five-valves shelf base.

Supplementary Table 4: Bill of materials for the SPPS Chemputer frame and shelving assembly.

Description	Manufacturer's part number	Amount	Supplier
90 Degree Joining Plate black anodized	VSLOT-M-P-90-5-B	4	Ooznest
Hex socket head cap screw M5x0.80 x 25 A2 Stainless Steel	DIN-912_M5X25_A2	12	
Hex socket head cap screw M5x0.80 x 8 A2 Stainless Steel	DIN-912_M5X8_A2	80	
Shelf base for 3 Chemputer pumps/valves	Built in house	1	N/A
Shelf base for 5 Chemputer pumps/valves	Built in house	2	N/A
Shelf for 3 Chemputer pumps	Built in house	2	N/A
Shelf for 5 Chemputer valves	Built in house	4	N/A
T-nut 6 mm slot M5	VSLOT-H-DT-M5	92	Ooznest
T/V-Slot 6mm Aluminium Extrusion Profile 20x20mm black anodized	AEP-TV-2020-B-CTS[L350]	2	Ooznest
T/V-Slot 6mm Aluminium Extrusion Profile 20x20mm black anodized	AEP-TV-2020-B-CTS[L490]	4	Ooznest
T/V-Slot 6mm Aluminium Extrusion Profile 20x20mm black anodized	AEP-TV-2020-B-CTS[L510]	6	Ooznest
T/V-Slot 6mm Aluminium Extrusion Profile 20x20mm black anodized	AEP-TV-2020-B-CTS[L80]	2	Ooznest
Universal L Bracket single black anodized	VSLOT-B-UL-S-B	30	Ooznest

3.3.3 SPPS reactor custom glassware

In addition to the previously reported glassware[4], a custom filter frit reactor (SPPS reactor; Supplementary Fig. 5) was designed to carry out solid-phase peptide assembly and resin cleavage in full automation on the SPPS Chemputer. Taking advantage of the versatility of a round-bottom flask (RBF), standard joint sizing (NS 24/29), fritted filter disc (of porosity P4), and the Diba Omnifit® adapter with 1/4"-28 UNF(M) connection used in Chemputer glassware, the SPPS reactor allowed for efficient automated peptide synthesis on the Chemputer.

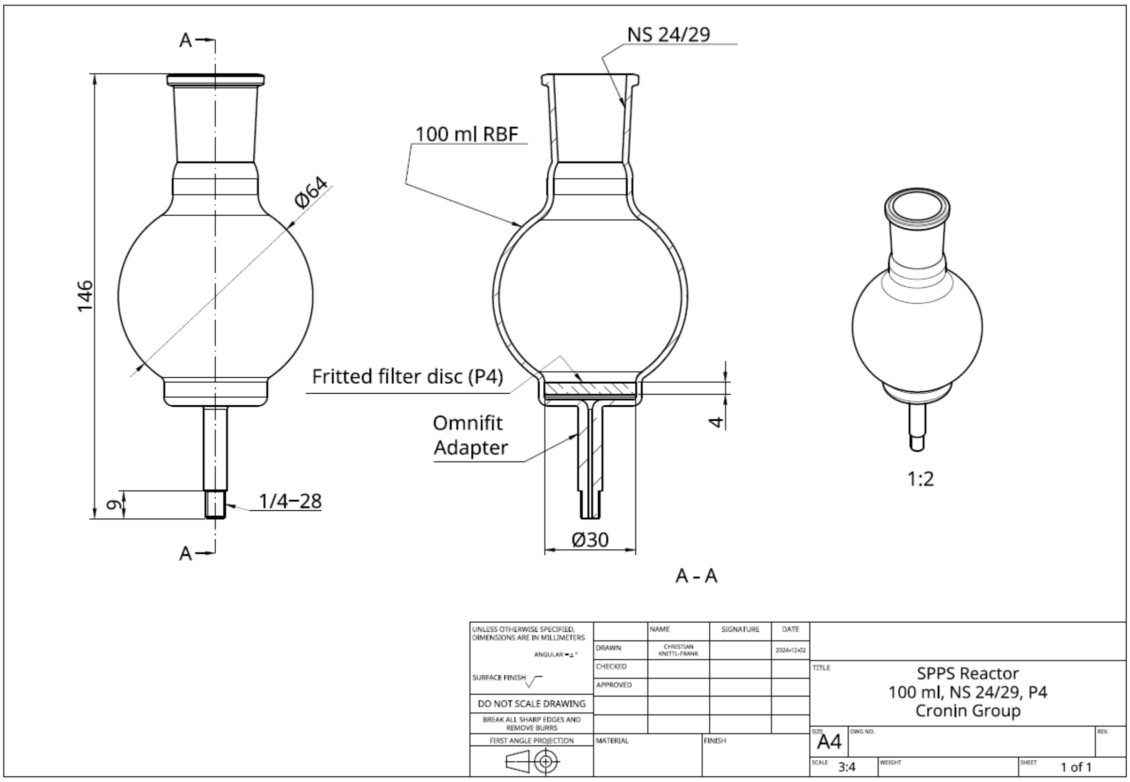

Supplementary Fig. 5: Technical drawing for the 100 mL SPPS reactor highlighting the general dimensions of the flask and the fritted filter disc.

4. χDL blueprints

The general synthetic protocol for SPPS was captured in unambiguous χDL blueprints, each representing one stage of the iterative cycle. The complete SPPS blueprint steps, in human-readable format, showcasing the sequential χDL unit operations executed by the Chemputer platform were the following:

Note: some steps are executed in parallel without waiting for the end of the step preceding them. These are highlighted by an asterisk (*) after the step index.

Note: the general blueprints showcased here contain universal values, highlighted in **bold**, for parameters regarding solvents, substrates, time, and more synthetic values. During execution, these are specified into the desired property in the χDL file.

Note: in the case a step required manual intervention (i.e. solid additions), this is highlighted in *italics*.

Note: the default speed used throughout this project is 40 mL/min.

Blueprint: Resin_swell

Step 1: Reset liquid handling apparatus with DMF (3 x 3 mL).

Step 2: Add DMF (9 mL) directly to spps_reactor at default speed.

Step 3: Purge spps_reactor with inert gas for 60 min.

Step 4: Filter contents of spps_reactor, applying vacuum for 50 s.

Blueprint: Deprotection

Step 1: Add 20% piperidine in DMF (9 mL) directly to spps_reactor at default speed.

Step 2: Purge spps_reactor with inert gas for 3 min.

Step 3: Filter contents of spps_reactor, applying vacuum for 30 s.

Step 4: Add 20% piperidine in DMF (9 mL) directly to spps_reactor at default speed.

Step 5*: Purge spps_reactor with inert gas for 12 min.

Step 6*: Reset liquid handling apparatus with DMF (3 x 3 mL).

Step 7: Filter contents of spps_reactor, applying vacuum for 50 s.

Blueprint: Resin_wash

Step 1: Add **wash_solvent** (9 mL) directly to spps_reactor at default speed.

Step 2: Purge spps_reactor with inert gas for 45 s.

Step 3: Filter contents of spps_reactor, applying vacuum for 30 s.

Step 4: Repeat 3 times:

Add **wash_solvent** (9 mL) directly to spps_reactor at default speed.

Purge spps_reactor with inert gas for 45 s.

Filter contents of spps_reactor, applying vacuum for 30 s.

Step 5: Add **wash_solvent** (9 mL) directly to spps_reactor at default speed.

Step 6: Purge spps_reactor with inert gas for 45 s.

Step 7: Filter contents of spps_reactor, applying vacuum for 50 s.

The parameter **wash_solvent** specifies the solvent to use when washing the resin solid support during the different stages of the synthesis. The set default value is DMF, meaning that if no other solvent is specified, DMF will be used.

Blueprint: Coupling

Step 1: **Deprotection**

Step 2: **Resin_wash**

Step 3: Repeat **coupling_repeats** times:

Add **amino_acid** (2 mL) directly to spps_reactor at default speed.

Add HATU (2 mL) directly to spps_reactor at default speed.

Add DIPEA (0.5 mL) directly to spps_reactor at default speed.

Purge* spps_reactor with inert gas for **coupling_time**.

Reset* liquid handling apparatus with DMF (3 x 3 mL).

Filter contents of spps_reactor, applying vacuum for 30 s.

Step 4: **Resin_wash**

The parameter **coupling_repeats** refers to how many times a single amino acid coupling procedure is repeated. The set default value is one, meaning that if no other value is specified, amino acid coupling will be executed one time.

The parameter **amino_acid** refers to the amino acid used for this particular coupling step. A unique value must be assigned to each **Coupling** blueprint step as this defines the synthetic operations for peptide synthesis. No default value is given.

The parameter **coupling_time** refers to the time employed for coupling of an amino acid. Specifically, it determines how long the mixture is sparged during the coupling step. The set default value is 30 min, meaning that if no other value is specified, amino acid coupling will be executed for 30 min.

Blueprint: Cleavage_and_workup

Step 1: Reset liquid handling apparatus with DCM (6 x 3 mL).

Step 2: **Resin_wash** – **wash_solvent** = DCM

Step 3: Dry contents of spps_reactor for 15 min at default pressure.

Step 4: Add TIPS (0.5 mL) directly to spps_reactor at 5 mL/min.

Step 5: Add H_2O (0.5 mL) directly to spps_reactor at 5 mL/min.

Step 6: Add TFA (9 mL) directly to spps_reactor at 20 mL/min.

Step 7*: **Cleavage_mix** for a total of 2 h.

Step 8*: Heat/Chill precipitating_unit to -20 °C without stirring. Temperature control is continued after the temperature has been reached.

Step 9*: Reset liquid handling apparatus with H_2O (3 x 3 mL).

Step 10*: Reset liquid handling apparatus with Et_2O (3 x 3 mL).

Step 11*: Add Et_2O (180 mL) directly to precipitating_unit at default speed.

Step 12: Transfer all from spps_reactor directly to precipitating_unit at 20 mL/min, flushing tubing after the transfer.

Step 13: Add TFA (10 mL) directly to spps_reactor at 20 mL/min.

Step 14: Purge spps_reactor with inert gas for 60 s.

Step 15: Transfer all from spps_reactor directly to precipitating_unit at 20 mL/min, flushing tubing after the transfer.

Step 16*: Purge precipitating_unit with inert gas for 30 min.

Step 17*: Reset liquid handling apparatus with H_2O (3 x 3 mL).

Step 18*: Reset liquid handling apparatus with Et_2O (3 x 3 mL).

Step 19: Filter contents of precipitating_unit, applying vacuum for 30 s, sending filtrate to supernatant using standard transfer speed.

Step 20: Repeat 3 times:

Add Et_2O (30 mL) directly to precipitating_unit at default speed.

Purge precipitating_unit with inert gas for 5 min.

Filter contents of precipitating_unit, applying vacuum for 30 s, sending filtrate to supernatant using standard transfer speed.

Step 21: Dry contents of precipitating_unit for 10 min at default pressure.

Step 22*: Heat/Chill precipitating_unit to 20 °C without stirring. Temperature control is stopped once the temperature has been reached.

Step 23*: Reset liquid handling apparatus with **peptide_solvent** (3 x 3 mL).

Step 24: Add **peptide_solvent** (20 mL) directly to precipitating_unit at default speed.

Step 25: Purge precipitating_unit with inert gas for 15 min.

Step 26: Transfer all from precipitating_unit directly to **collection_flask** at default speed, flushing tubing after the transfer.

Step 27: Repeat 2 times:

Add **peptide_solvent** (10 mL) directly to precipitating_unit at default speed.

Purge precipitating_unit with inert gas for 2 min.

Transfer all from precipitating_unit directly to **collection_flask** at default speed, flushing tubing after the transfer.

Step 28: Reset liquid handling apparatus with DCM (3 x 3 mL).

Step 29: Reset liquid handling apparatus with DMF (3 x 3 mL).

The blueprint step **Cleavage_mix** is a simple blueprint to allow the gentle sparging of the resin in the cleavage mixture and the parallel execution of the following steps in preparation of peptide precipitation and workup.

The parameter **peptide_solvent** refers to the solvent used to dissolve the peptide after ether workup. This would usually be MeCN/H2O (50:50 v/v) for lyophilization, an adequate solvent for chemical modifications, or a buffer solution as required.

The parameter **collection_flask** refers to the destination vessel after diethyl ether workup. This can be a vial for subsequent lyophilization, a reactor for chemical modifications, or another module as required.

Blueprint Acetylation:

Step 1: **Deprotection**

Step 2: **Resin_wash**

Step 3: Repeat **capping_repeats** times:

Add Ac_2O in DMF (9.5 mL) directly to spps_reactor at default speed.

Add DIPEA (0.5 mL) directly to spps_reactor at default speed.

Purge spps_reactor with inert gas for **capping_time**.

Filter contents of spps_reactor, applying vacuum for 50 s.

Step 4: Reset liquid handling apparatus with DMF (3 x 3 mL).

Step 5: **Resin_wash.**

The parameter **capping_repeats** refers to the number of times the capping procedure is repeated. The default value is set to one.

The parameter **capping_time** refers to the amount of time the capping mixture is purged during the procedure. The default value is set to 3 min.

Blueprint: Mtt_deprotection

Step 1: Reset liquid handling apparatus with DCM (3 x 3 mL).

Step 2: **Resin_wash** – **wash_solvent** = DCM

Step 3: Repeat **deprotection_repeats** times:

Add DCM (2.5 mL) directly to spps_reactor at default speed.

Add HFIP (7 mL) directly to spps_reactor at default speed.

Add TIPS (0.5 mL) directly to spps_reactor at 5 mL/min.

Cleavage_mix* for a total of 30 min.

Reset* liquid handling apparatus with DCM (3 x 3 mL).

Filter contents of spps_reactor, applying vacuum for 30 s.

Step 4: **Resin_wash** – **wash_solvent** = DCM.

The parameter **deprotection_repeats** refers to the number of times the deprotection procedure is repeated. The default value is set to one.

Blueprint: No_deprotection_substrate_coupling

Step 1: **Resin_wash**

Step 2: Repeat **coupling_repeats** times:

Add **substrate** (2 mL) directly to spps_reactor at default speed.

Add HATU (2 mL) directly to spps_reactor at default speed.

Add DIPEA (0.5 mL) directly to spps_reactor at default speed.

Purge* spps_reactor with inert gas for **coupling_time**.

Reset* liquid handling apparatus with DMF (3 x 3 mL).

Filter contents of spps_reactor, applying vacuum for 30 s.

Step 3: **Resin_wash**

The parameter **substrate** refers to the molecular substrate used for this particular coupling step. A unique value must be assigned to each blueprint step as this defines the synthetic operations for this specific coupling. No default value is given.

Blueprint: Substrate_coupling

Step 1: **Deprotection**

Step 2: **Resin_wash**

Step 3: Repeat **coupling_repeats** times:

Add **substrate** (2 mL) directly to spps_reactor at default speed.

Add HATU (2 mL) directly to spps_reactor at default speed.

Add DIPEA (0.5 mL) directly to spps_reactor at default speed.

Purge* spps_reactor with inert gas for **coupling_time**.

Reset* liquid handling apparatus with DMF (3 x 3 mL).

Filter contents of spps_reactor, applying vacuum for 30 s.

Step 4: **Resin_wash**

Blueprint: RCM

Step 1: Set stir rate of M102_flask_1 to 250 RPM.

Step 2: Set stir rate of M102_flask_2 to 250 RPM.

Step 3: Reset liquid handling apparatus with DCM (3 x 3 mL).

Step 4: **Resin_wash** – **wash_solvent** = DCM

Step 5: Reset liquid handling apparatus with DCE (3 x 3 mL).

Step 6: **Resin_wash** – **wash_solvent** = DCE

Step 7: Add DCE (7 mL) directly to M102_flask_1 at default speed.

Step 8: Start purging M102_flask_1 with inert gas.

Step 9: Stir M102_flask_1 for 60 s at 250 RPM stopping stirring afterwards.

Step 10: Stop purging M102_flask_1 with inert gas.

Step 11: Transfer all from M102_flask_1 directly to spps_reactor at 20 mL/min, flushing tubing after the transfer.

Step 12*: Purge spps_reactor with inert gas for 2 h.

Step 13*: Reset liquid handling apparatus with DCE (3 x 3 mL).

Step 14: Filter contents of spps_reactor, applying vacuum for 50 s.

Step 15: Add DCE (7 mL) directly to M102_flask_2 at default speed.

Step 16: Start purging M102_flask_2 with inert gas.

Step 17: Stir M102_flask_2 for 60 s at 250 RPM stopping stirring afterwards.

Step 18: Stop purging M102_flask_2 with inert gas.

Step 19: Transfer all from M102_flask_2 directly to spps_reactor at 20 mL/min, flushing tubing after the transfer.

Step 20*: Purge spps_reactor with inert gas for 2 h.

Step 21*: Reset liquid handling apparatus with DCE (3 x 3 mL).

Step 22: Filter contents of spps_reactor, applying vacuum for 50 s.

Step 23: **Resin_wash** – **wash_solvent** = DCE

Step 24: **Resin_wash** – **wash_solvent** = DCM

Step 25: **Resin_wash** – **wash_solvent** = DMF

Blueprint: Cleavage_workup_click

Step 1: Set stir rate of reactor_1 to 250 RPM.

Step 2: Reset liquid handling apparatus with DCM (6 x 3 mL).

Step 3: **Resin_wash** – **wash_solvent** = DCM

Step 4: Dry contents of spps_reactor for 15 min at default pressure.

Step 5: Add TIPS (0.5 mL) directly to spps_reactor at 5 mL/min.

Step 6: Add H_2O (0.5 mL) directly to spps_reactor at 5 mL/min.

Step 7: Add TFA (9 mL) directly to spps_reactor at 20 mL/min.

Step 8*: **Cleavage_mix** for a total of 2 h.

Step 9*: Heat/Chill precipitating_unit to -20 °C without stirring. Temperature control is continued after the temperature has been reached.

Step 10*: Reset liquid handling apparatus with H_2O (3 x 3 mL).

Step 11*: Reset liquid handling apparatus with Et_2O (3 x 3 mL).

Step 12*: Add Et_2O (180 mL) directly to precipitating_unit at default speed.

Step 13: Transfer all from spps_reactor directly to precipitating_unit at 20 mL/min, flushing tubing after the transfer.

Step 14: Add TFA (10 mL) directly to spps_reactor at 20 mL/min.

Step 15: Purge spps_reactor with inert gas for 60 s.

Step 16: Transfer all from spps_reactor directly to precipitating_unit at 20 mL/min, flushing tubing after the transfer.

Step 17*: Purge precipitating_unit with inert gas for 30 min.

Step 18*: Reset liquid handling apparatus with H_2O (3 x 3 mL).

Step 19*: Reset liquid handling apparatus with Et_2O (3 x 3 mL).

Step 20: Filter contents of precipitating_unit, applying vacuum for 30 s, sending filtrate to supernatant using standard transfer speed.

Step 21: Repeat 3 times:

> Add Et_2O (30 mL) directly to precipitating_unit at default speed.
>
> Purge precipitating_unit with inert gas for 5 min.
>
> Filter contents of precipitating_unit, applying vacuum for 30 s, sending filtrate to supernatant using standard transfer speed.

Step 22: Dry contents of precipitating_unit for 10 min at default pressure.

Step 23*: Heat/Chill precipitating_unit to 20 °C without stirring. Temperature control is stopped once the temperature has been reached.

Step 24*: Reset liquid handling apparatus with **peptide_solvent** (3 x 3 mL).

Step 25: Add **peptide_solvent** (20 mL) directly to precipitating_unit at default speed.

Step 26: Purge precipitating_unit with inert gas for 15 min.

Step 27: Transfer all from precipitating_unit directly to **collection_flask** at default speed, flushing tubing after the transfer.

Step 28: Repeat 2 times:

> Add **peptide_solvent** (12.5 mL) directly to precipitating_unit at default speed.
>
> Purge precipitating_unit with inert gas for 2 min.
>
> Transfer all from precipitating_unit directly to **collection_flask** at default speed, flushing tubing after the transfer.

Step 29*: Reset liquid handling apparatus with DMF (3 x 3 mL).

Step 30*: Purge **collection_flask** with inert gas for 10 min.

Step 31*: Stir **collection_flask** for 12 h at 500 RPM stopping stirring afterwards.

Blueprint: Peptide_stapling_diversification

Step 1: Set stir rate of reactor_1 to 250 RPM.

Step 2: Add linear_peptide (6 mL) directly to reactor_1 at default speed.

Step 3: Add linear_peptide (6 mL) directly to reactor_2 at default speed.

Step 4: Add linear_peptide (6 mL) directly to reactor_3 at default speed.

Step 5: Add linear_peptide (6 mL) directly to reactor_4 at default speed.

Step 6: Reset liquid handling apparatus with DMF (3 x 3 mL).

Step 7: Add Hexafluorobenzene (4 mL) directly to reactor_1 at default speed.

Step 8: Add TRIS (5 mL) directly to reactor_1 at default speed.

Step 9: Reset liquid handling apparatus with DMF (3 x 3 mL).

Step 10: Add Decafluorobiphenyl (4 mL) directly to reactor_2 at default speed.

Step 11: Add TRIS (5 mL) directly to reactor_2 at default speed.

Step 12: Reset liquid handling apparatus with DMF (3 x 3 mL).

Step 13: Add 1,3-Bis(pentafluorophenyl)urea (4 mL) directly to reactor_3 at default speed.

Step 14: Add TRIS (5 mL) directly to reactor_3 at default speed.

Step 15: Reset liquid handling apparatus with DMF (3 x 3 mL).

Step 16: Add Tris(pentafluorophenyl)phosphine (4 mL) directly to reactor_4 at default speed.

Step 17: Add TRIS (5 mL) directly to reactor_4 at default speed.

Step 18: Reset liquid handling apparatus with DMF (3 x 3 mL).

Step 19: Stir reactor_1 for 12 h at 250 RPM stopping stirring afterwards.

Blueprint: OPA_cyclization

Step 1: Set stir rate of reactor_1 to 250 RPM.

Step 2: Set stir rate of flask_OPA to 250 RPM.

Step 3: Reset liquid handling apparatus with H_2O (3 x 3 mL).

Step 4: Add PBS Buffer (80 mL) directly to reactor_1 at default speed with stirring at 250 RPM.

Step 5: Reset liquid handling apparatus with DMSO (3 x 3 mL).

Step 6: Add DMSO (5 mL) directly to flask_OPA at 5 mL/min with stirring at 250 RPM.

Step 7: Stir flask_OPA for 2 min at 250 RPM stopping stirring afterwards.

Step 8: Add OPA_solution (1 mL) directly to reactor_1 over 15 min with stirring at 250 RPM.

Step 9*: Stir reactor_1 for 30 min at 250 RPM stopping stirring afterwards.

Step 10*: Reset liquid handling apparatus with DMSO (3 x 3 mL).

Blueprint: Maleimide_derivatization

Step 1: Set stir rate of reactor_1 to 250 RPM.

Step 2: Set stir rate of flask_Mal-FAM to 250 RPM.

Step 3: Add DMSO (4 mL) directly to flask_Mal-FAM at 5 mL/min with stirring at 250 RPM.

Step 4: Stir flask_Mal-FAM for 2 min at 250 RPM stopping stirring afterwards.

Step 5: Add Mal-FAM_solution (3.5 mL) directly to reactor_1 over 15 min with stirring at 250 RPM.

Step 6*: Stir reactor_1 for 30 min at 250 RPM stopping stirring afterwards.

Step 7*: Reset liquid handling apparatus with DMSO (3 x 3 mL).

Step 8*: Reset liquid handling apparatus with DMF (3 x 3 mL).

Blueprint: Resin_loading

Step 1: Repeat **loading_repeats** times:

Add **loading_reagent** (2 mL) directly to spps_reactor at default speed.

Purge* spps_reactor with inert gas for **loading_time**.

Reset* liquid handling apparatus with DMF (3 x 3 mL).

Filter contents of spps_reactor, applying vacuum for 30 s.

Step 2: **Resin_wash**

The parameter **loading_repeats** refers to how many times the coupling of the loading reagent to the resin is repeated. The set default value is one.

The parameter **loading_reagent** refers to the reagent to be loaded onto the resin. A unique value must be assigned to each **Resin_loading** blueprint step as this defines the synthetic operations for this synthesis. No default value is given.

The parameter **loading_time** refers to the time employed for loading of a reagent onto the resin. Specifically, it determines how long the mixture is sparged during the loading step. The set default value is 30 min.

Blueprint: Cleavage_and_workup_for_NCL

Step 1: Reset liquid handling apparatus with DCM (6 x 3 mL).

Step 2: **Resin_wash** – **wash_solvent** = DCM

Step 3: Dry contents of spps_reactor for 15 min at default pressure.

Step 4: Add TIPS (0.5 mL) directly to spps_reactor at 5 mL/min.

Step 5: Add H_2O (0.5 mL) directly to spps_reactor at 5 mL/min.

Step 6: Add TFA (9 mL) directly to spps_reactor at 20 mL/min.

Step 7*: **Cleavage_mix** for a total of 2 h.

Step 8*: Heat/Chill precipitating_unit to -20 °C without stirring. Temperature control is continued after the temperature has been reached.

Step 9*: Reset liquid handling apparatus with H_2O (3 x 3 mL).

Step 10*: Reset liquid handling apparatus with Et_2O (3 x 3 mL).

Step 11*: Add Et_2O (180 mL) directly to precipitating_unit at default speed.

Step 12: Transfer all from spps_reactor directly to precipitating_unit at 20 mL/min, flushing tubing after the transfer.

Step 13: Add TFA (10 mL) directly to spps_reactor at 20 mL/min.

Step 14: Purge spps_reactor with inert gas for 60 s.

Step 15: Transfer all from spps_reactor directly to precipitating_unit at 20 mL/min, flushing tubing after the transfer.

Step 16*: Purge precipitating_unit with inert gas for 30 min.

Step 17*: Reset liquid handling apparatus with H_2O (3 x 3 mL).

Step 18*: Reset liquid handling apparatus with Et_2O (3 x 3 mL).

Step 19: Filter contents of precipitating_unit, applying vacuum for 30 s, sending filtrate to supernatant using standard transfer speed.

Step 20: Repeat 3 times:

Add Et_2O (30 mL) directly to precipitating_unit at default speed.

Purge precipitating_unit with inert gas for 5 min.

Filter contents of precipitating_unit, applying vacuum for 30 s, sending filtrate to supernatant using standard transfer speed.

Step 21: Dry contents of precipitating_unit for 10 min at default pressure.

Step 22*: Heat/Chill precipitating_unit to 20 °C without stirring. Temperature control is stopped once the temperature has been reached.

Step 23*: Reset liquid handling apparatus with **peptide_solvent** (3 x 3 mL).

Step 24: Add **peptide_solvent** (20 mL) directly to precipitating_unit at default speed.

Step 25: Purge precipitating_unit with inert gas for 15 min.

Step 26: Transfer all from precipitating_unit directly to **collection_flask** at default speed, flushing tubing after the transfer.

Step 27: Repeat 2 times:

> Add **peptide_solvent** (8.75 mL) directly to precipitating_unit at default speed.
>
> Purge precipitating_unit with inert gas for 2 min.
>
> Transfer all from precipitating_unit directly to **collection_flask** at default speed, flushing tubing after the transfer.

Step 28: Reset liquid handling apparatus with DCM (3 x 3 mL).

Step 29: Reset liquid handling apparatus with H_2O (3 x 3 mL).

Blueprint: Thioesterification_and_NCL

Step 1: Set stir rate of **collection_flask** to 250 RPM.

Step 2: Set stir rate of reactor_2 to 250 RPM.

Step 3: Add Acac (0.743 mL) directly to **collection_flask** at default speed with stirring at 250 RPM.

Step 4*: Stir **collection_flask** for 4 h at 250 RPM stopping stirring afterwards.

Step 5*: Reset liquid handling apparatus with H_2O (3 x 3 mL).

Step 6: Transfer all from reactor_2 directly to waste_21 at default speed, flushing tubing after the transfer.

Step 7: Reset liquid handling apparatus with H_2O (3 x 3 mL).

Step 8: Repeat 2 times:

> Add H_2O (100 mL) directly to reactor_2 at default speed with stirring at 250 RPM.
>
> Stir reactor_2 for 60 s at 250 RPM stopping stirring afterwards.
>
> Transfer all from reactor_2 directly to waste_21 at default speed, flushing tubing after the transfer.

Step 9: Add Buffer B (82.5 mL) directly to reactor_2 at default speed with stirring at 250 RPM.

Step 10: Reset liquid handling apparatus with H_2O (3 x 3 mL).

Step 11: Transfer all from **collection_flask** directly to reactor_2 at default speed, flushing tubing after the transfer.

Step 12: Repeat 2 times:

> Add Buffer B (5 mL) directly to **collection_flask** at default speed with stirring at 250 RPM.
>
> Stir **collection_flask** for 60 s at 250 RPM stopping stirring afterwards.
>
> Transfer all from **collection_flask** directly to reactor_2 at default speed, flushing tubing after the transfer.

Step 13: Reset liquid handling apparatus with H_2O (3 x 3 mL).

Step 14: Set stir rate to 250 RPM and start stirring reactor_2.

Step 15: Move NaOH (3 mL) to waste in order to prime pump.

Step 16: Repeat until done:

> Add NaOH (0.25 mL) directly to reactor_2 at default speed with stirring at 250 RPM.
>
> Stir reactor_2 for 60 s at 250 RPM leaving stirring on afterwards.
>
> Monitor pH of reactor_2.

Step 17: Reset liquid handling apparatus with H_2O (3 x 3 mL).

Step 18: Move HCl (3 mL) to waste in order to prime pump.

Step 19: Repeat until done:

> Add HCl (0.125 mL) directly to reactor_2 at default speed with stirring at 250 RPM.
>
> Stir reactor_2 for 60 s at 250 RPM leaving stirring on afterwards.
>
> Monitor pH of reactor_2.

Step 20: Transfer all from reactor_2 directly to **collection_flask** at default speed, flushing tubing after the transfer.

Step 21: Repeat 2 times:

> Add Buffer B (5 mL) directly to reactor_2 at default speed.
>
> Stir reactor_2 for 60 s at 250 RPM stopping stirring afterwards.
>
> Transfer all from reactor_2 directly to **collection_flask** at default speed, flushing tubing after the transfer.

Step 22*: Stir **collection_flask** for 12 h at 250 RPM stopping stirring afterwards.

Step 23*: Repeat 3 times:

Add H_2O (50 mL) directly to reactor_2 at default speed with stirring at 250 RPM.

Stir reactor_2 for 60 s at 250 RPM stopping stirring afterwards.

Transfer all from reactor_2 directly to waste_21 at default speed, flushing tubing after the transfer.

Step 24*: Add KCl (100 mL) directly to reactor_2 at default speed.

Step 25*: Reset liquid handling apparatus with H_2O (3 x 3 mL).

5. Fully automated synthesis of peptides

5.1 ACP(65-74) (1)

Linear sequence: H-VQAAIDYING-OH (10 aa) (**1**)
Resin: Fmoc-Gly-Wang (0.36 mmol/g)
Crude purity: 90% by RP-HPLC at 214 nm
Crude yield: 53 mg (41%)
Steps: 22 chemical steps, 497-unit operations
Synthesis time: 20:49 (hh:mm)

The χDL file **1_ACP(65-74).xdl** and the graph file **1_ACP(65-74).json** were executed as described above. Blueprint output steps resulting from the χDL execution were as follows:

Step 1: Add Fmoc-Gly-Wang resin (278 mg) directly to spps_reactor.
Step 2: Resin_swell
Step 3: Coupling – **amino_acid** = Asn
Step 4: Coupling – **amino_acid** = Ile
Step 5: Coupling – **amino_acid** = Tyr
Step 6: Coupling – **amino_acid** = Asp
Step 7: Coupling – **amino_acid** = Ile
Step 8: Coupling – **amino_acid** = Ala
Step 9: Coupling – **amino_acid** = Ala
Step 10: Coupling – **amino_acid** = Gln
Step 11: Coupling – **amino_acid** = Val
Step 12: Deprotection
Step 13: Resin_wash
Step 14: Cleavage_and_workup – **peptide_solvent** = MeCN/H_2O (50:50 v/v), **collection_flask** = product_flask
Step 15: Shut down the platform.

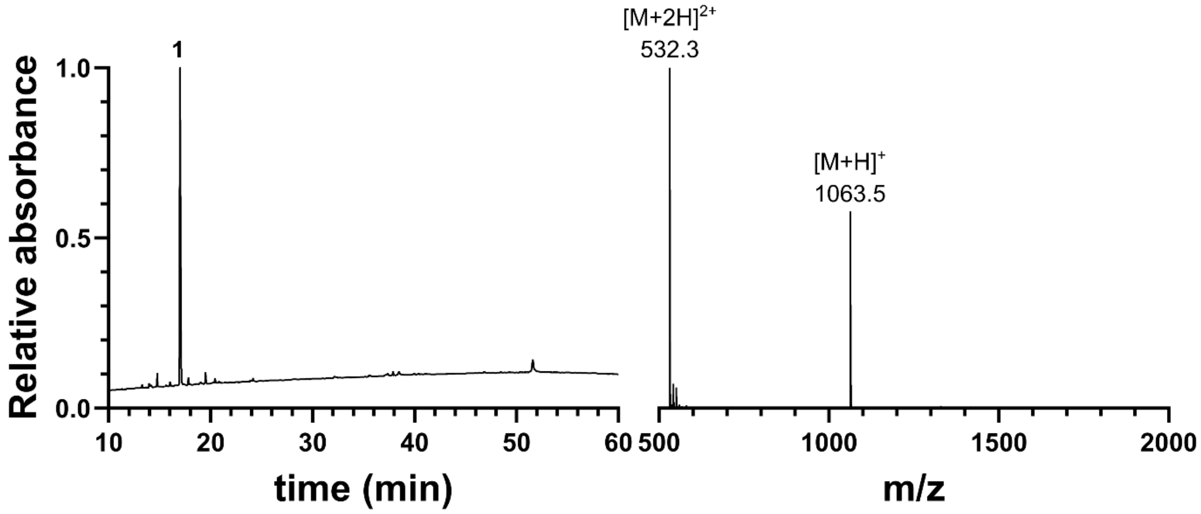

Supplementary Fig. 6: Crude RP-HPLC (214 nm) and ESI-MS analysis of ACP(65-74) (**1**).

5.2 18A (2)

**Linear sequence:**	Ac-DWLKAFYDKVAEKLKEAF-NH_2 (18 aa) (**2**)
**Resin:**	Fmoc-Rink Amide AM (0.42 mmol/g)
**Crude purity:**	93% by RP-HPLC at 214 nm
**Crude yield:**	195 mg (67%)
**Steps:**	40 chemical steps, 916-unit operations
**Synthesis time:**	34:26 (hh:mm)

The χDL file **2_18A.xdl** and the graph file **2_18A.json** were executed as described above. Blueprint output steps resulting from the χDL execution were as follows:

Step 1: *Add Fmoc-Rink Amide AM resin (238 mg) directly to spps_reactor.*
Step 2: Resin_swell
Step 3: Coupling – **amino_acid** = Phe
Step 4: Coupling – **amino_acid** = Ala
Step 5: Coupling – **amino_acid** = Glu
Step 6: Coupling – **amino_acid** = Lys
Step 7: Coupling – **amino_acid** = Leu
Step 8: Coupling – **amino_acid** = Lys
Step 9: Coupling – **amino_acid** = Glu
Step 10: Coupling – **amino_acid** = Ala
Step 11: Coupling – **amino_acid** = Val
Step 12: Coupling – **amino_acid** = Lys
Step 13: Coupling – **amino_acid** = Asp
Step 14: Coupling – **amino_acid** = Tyr
Step 15: Coupling – **amino_acid** = Phe
Step 16: Coupling – **amino_acid** = Ala
Step 17: Coupling – **amino_acid** = Lys
Step 18: Coupling – **amino_acid** = Leu
Step 19: Coupling – **amino_acid** = Trp
Step 20: Coupling – **amino_acid** = Asp
Step 21: Acetylation – **capping_repeats** = 2
Step 22: Cleavage_and_workup – **peptide_solvent** = MeCN/H_2O (50:50 v/v), **collection_flask** = product_flask
Step 23: Shut down the platform.

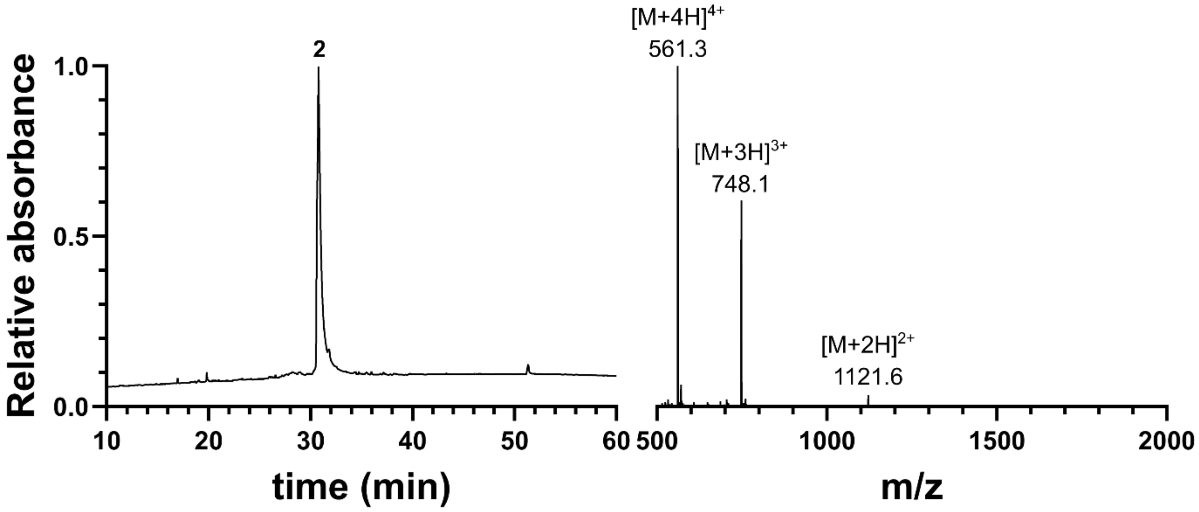

Supplementary Fig. 7: Crude RP-HPLC (214 nm) and ESI-MS analysis of 18A (**2**).

5.3 GHRH(1-29) (3)

Linear sequence: H-YADAIFTNSYRKVLGQLSARKLLQDILSA-NH_2 (29 aa) (**3**)
Resin: Fmoc-Rink Amide AM (0.42 mmol/g)
Crude purity: 87% by RP-HPLC at 214 nm
Crude yield: 261 mg (59%)
Steps: 62 chemical steps,1377-unit operations
Synthesis time: 49:55 (hh:mm)

The χDL file **3_GHRH(1-29).xdl** and the graph file **3_GHRH(1-29).json** were executed as described above. Blueprint output steps resulting from the χDL execution were as follows:

Step 1: Add Fmoc-Rink Amide AM resin (238 mg) directly to spps_reactor.
Step 2: Resin_swell
Step 3: Coupling – **amino_acid** = Ala
Step 4: Coupling – **amino_acid** = Ser
Step 5: Coupling – **amino_acid** = Leu
Step 6: Coupling – **amino_acid** = Ile
Step 7: Coupling – **amino_acid** = Asp
Step 8: Coupling – **amino_acid** = Gln
Step 9: Coupling – **amino_acid** = Leu
Step 10: Coupling – **amino_acid** = Leu
Step 11: Coupling – **amino_acid** = Lys
Step 12: Coupling – **amino_acid** = Arg
Step 13: Coupling – **amino_acid** = Ala
Step 14: Coupling – **amino_acid** = Ser
Step 15: Coupling – **amino_acid** = Leu
Step 16: Coupling – **amino_acid** = Gln
Step 17: Coupling – **amino_acid** = Gly
Step 18: Coupling – **amino_acid** = Leu
Step 19: Coupling – **amino_acid** = Val
Step 20: Coupling – **amino_acid** = Lys
Step 21: Coupling – **amino_acid** = Arg
Step 22: Coupling – **amino_acid** = Tyr
Step 23: Coupling – **amino_acid** = Ser
Step 24: Coupling – **amino_acid** = Asn
Step 25: Coupling – **amino_acid** = Thr
Step 26: Coupling – **amino_acid** = Phe
Step 27: Coupling – **amino_acid** = Ile
Step 28: Coupling – **amino_acid** = Ala
Step 29: Coupling – **amino_acid** = Asp
Step 30: Coupling – **amino_acid** = Ala
Step 31: Coupling – **amino_acid** = Tyr
Step 32: Deprotection
Step 33: Resin_wash
Step 34: Cleavage_and_workup – **peptide_solvent** = MeCN/H_2O (50:50 v/v), **collection_flask** = product_flask
Step 35: Shut down the platform.

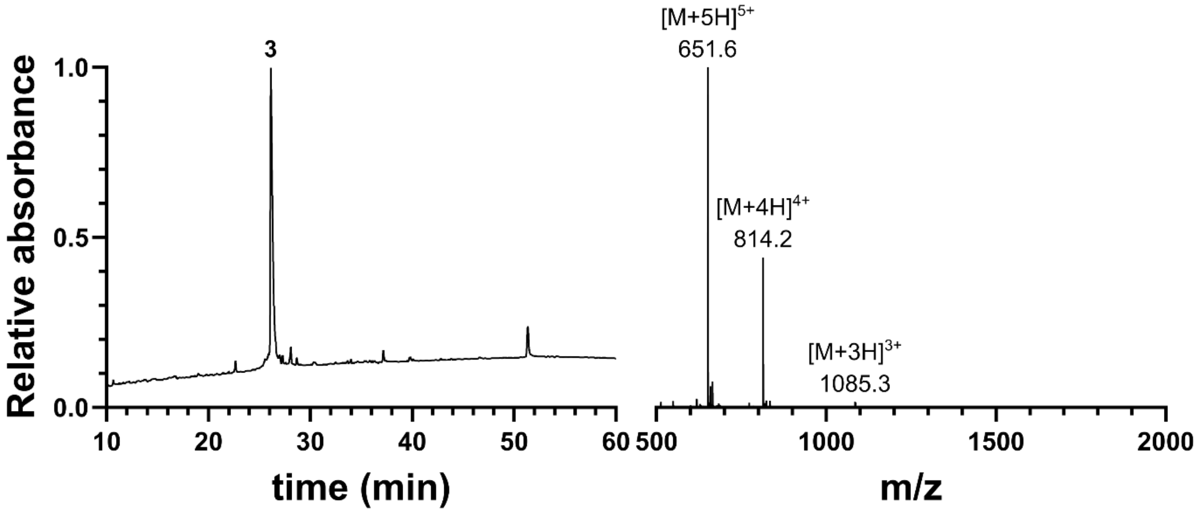

Supplementary Fig. 8: Crude RP-HPLC (214 nm) and ESI-MS analysis of GHRH(1-29) (**3**).

5.4 Semaglutide – on-resin sidechain functionalization (4)

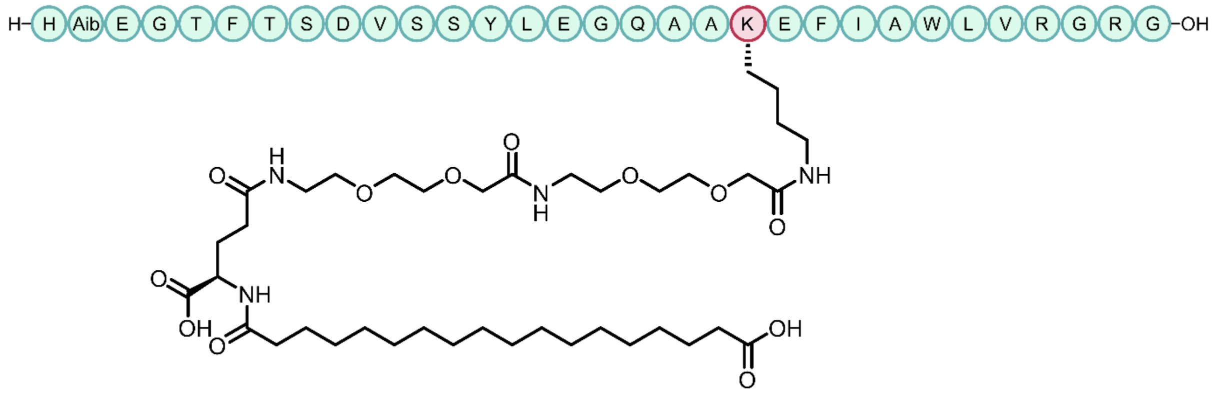

4

Linear sequence: H-H(Aib)EGTFTSDVSSYLEGQAAKEFIAWLVRGRG-OH (31 aa)
Resin: Fmoc-Gly-Wang (0.36 mmol/g)
Yield: 80 mg (18%)
Steps: 71 chemical steps (1635-unit operations)
Synthesis time: 70:04 (hh:mm)

The χDL file **4_Semaglutide.xdl** and the graph file **4_Semaglutide.json** were executed as described above. Blueprint output steps resulting from the χDL execution were as follows:

Step 1: *Add Fmoc-Gly-Wang resin (278 mg) directly to spps_reactor.*
Step 2: Resin_swell
Step 3: Coupling – **amino_acid** = Arg
Step 4: Coupling – **amino_acid** = Gly
Step 5: Coupling – **amino_acid** = Arg
Step 6: Coupling – **amino_acid** = Val
Step 7: Coupling – **amino_acid** = Leu
Step 8: Coupling – **amino_acid** = Trp
Step 9: Coupling – **amino_acid** = Ala
Step 10: Coupling – **amino_acid** = Ile
Step 11: Coupling – **amino_acid** = Phe
Step 12: Coupling – **amino_acid** = Glu
Step 13: Coupling – **amino_acid** = Lys(Mtt)
Step 14: Coupling – **amino_acid** = Ala
Step 15: Coupling – **amino_acid** = Ala
Step 16: Coupling – **amino_acid** = Gln
Step 17: Coupling – **amino_acid** = Gly
Step 18: Coupling – **amino_acid** = Glu
Step 19: Coupling – **amino_acid** = Leu
Step 20: Coupling – **amino_acid** = Tyr
Step 21: Coupling – **amino_acid** = Ser
Step 22: Coupling – **amino_acid** = Ser
Step 23: Coupling – **amino_acid** = Val
Step 24: Coupling – **amino_acid** = Asp
Step 25: Coupling – **amino_acid** = Ser
Step 26: Coupling – **amino_acid** = Thr
Step 27: Coupling – **amino_acid** = Phe
Step 28: Coupling – **amino_acid** = Thr
Step 29: Coupling – **amino_acid** = Gly
Step 30: Coupling – **amino_acid** = Glu

Step 31: Coupling – **amino_acid** = Aib
Step 32: Coupling – **amino_acid** = Boc-His
Step 33: Mtt_deprotection – **deprotection_repeats** = 2
Step 34: No_deprotection_substrate_coupling – **substrate** = Fmoc-Amino-3,6 dioxaoctanoic acid, **coupling_time** = 1 h, **coupling_repeats** = 2
Step 35: Substrate_coupling – **substrate** = Fmoc-Amino-3,6 dioxaoctanoic acid, **coupling_time** = 1 h, **coupling_repeats** = 2
Step 36: Coupling – **amino_acid** = Fmoc-Glu-O*t*Bu, **coupling_time** = 1 h, **coupling_repeats** = 2
Step 37: Substrate_coupling – **substrate** = 1,18-octadecanedioic acid, **coupling_time** = 2 h, **coupling_repeats** = 2
Step 38: Cleavage_and_workup – **peptide_solvent** = MeCN/H_2O (50:50 v/v), **collection_flask** = product_flask
Step 39: Shut down the platform.

The digital procedure was translated into the executed χDL file from literature reported methods[5,6].

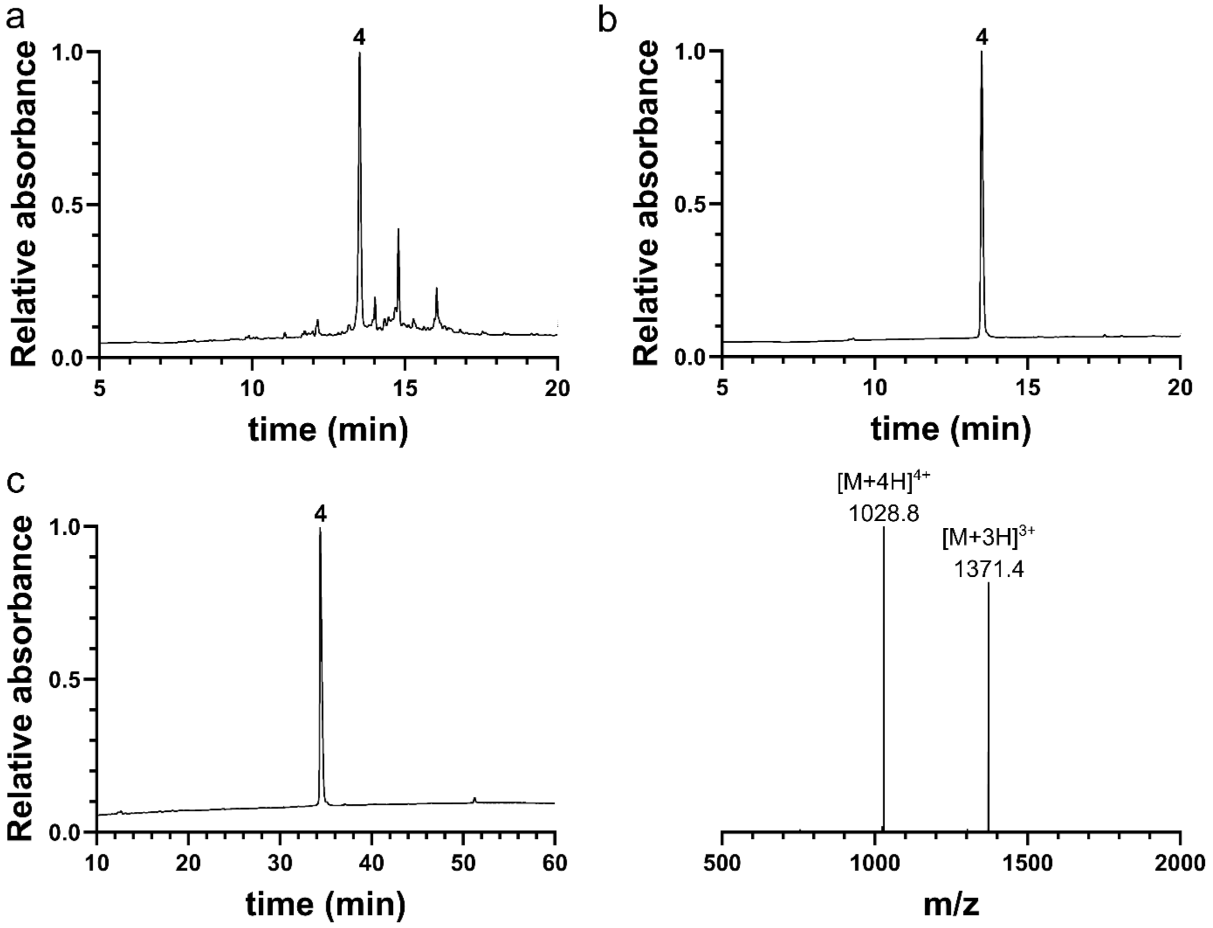

Supplementary Fig. 9: RP-HPLC traces and ESI-MS of Semaglutide (**4**). **a**, Crude RP-HPLC trace (214 nm, 19 min 0–80% MeCN gradient). **b**, Purified RP-HPLC trace (214 nm, 19 min 0–80% MeCN gradient). **c**, Purified RP-HPLC trace (214 nm, 60 min 0–80% MeCN gradient) and ESI-MS.

5.5 NYAD-13 – on-resin ring-closing metathesis (5)

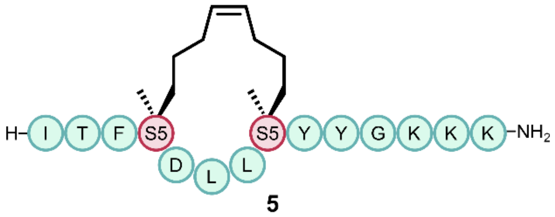

5

Linear sequence: H-ITF(S5)DLL(S5)YYGKKK-NH_2 (14 aa)
Resin: Fmoc-Rink Amide AM (0.42 mmol/g)
Yield: 138 mg (63%)
Steps: 32 chemical steps (823-unit operations)
Synthesis time: 33:53 (hh:mm)

The χDL file **5_NYAD-13.xdl** and the graph file **5_NYAD-13.json** were executed as described above. Blueprint output steps resulting from the χDL execution were as follows:

Step 1: *Add Fmoc-Rink Amide AM resin (238 mg) directly to spps_reactor.*
Step 2: *Add M102 (58 mg) directly to M102_flask_1.*
Step 3: *Add M102 (58 mg) directly to M102_flask_2.*
Step 4: Set stir rate of M102_flask_1 to 250 RPM.
Step 5: Set stir rate of M102_flask_2 to 250 RPM.
Step 6: Resin_swell
Step 7: Coupling – **amino_acid** = Lys
Step 8: Coupling – **amino_acid** = Lys
Step 9: Coupling – **amino_acid** = Lys
Step 10: Coupling – **amino_acid** = Gly
Step 11: Coupling – **amino_acid** = Tyr
Step 12: Coupling – **amino_acid** = Tyr
Step 13: Coupling – **amino_acid** = S5, **coupling_time** = 1 h
Step 14: Coupling – **amino_acid** = Leu
Step 15: Coupling – **amino_acid** = Leu
Step 16: Coupling – **amino_acid** = Asp
Step 17: Coupling – **amino_acid** = S5, **coupling_time** = 1 h
Step 18: Coupling – **amino_acid** = Phe
Step 19: Coupling – **amino_acid** = Thr
Step 20: Coupling – **amino_acid** = Ile
Step 21: RCM
Step 22: Deprotection
Step 23: Resin_wash
Step 24: Cleavage_and_workup – **peptide_solvent** = MeCN/H_2O (50:50 v/v), **collection_flask** = product_flask
Step 23: Shut down the platform.

The digital procedure was translated into the executed χDL file from literature reported methods[7].

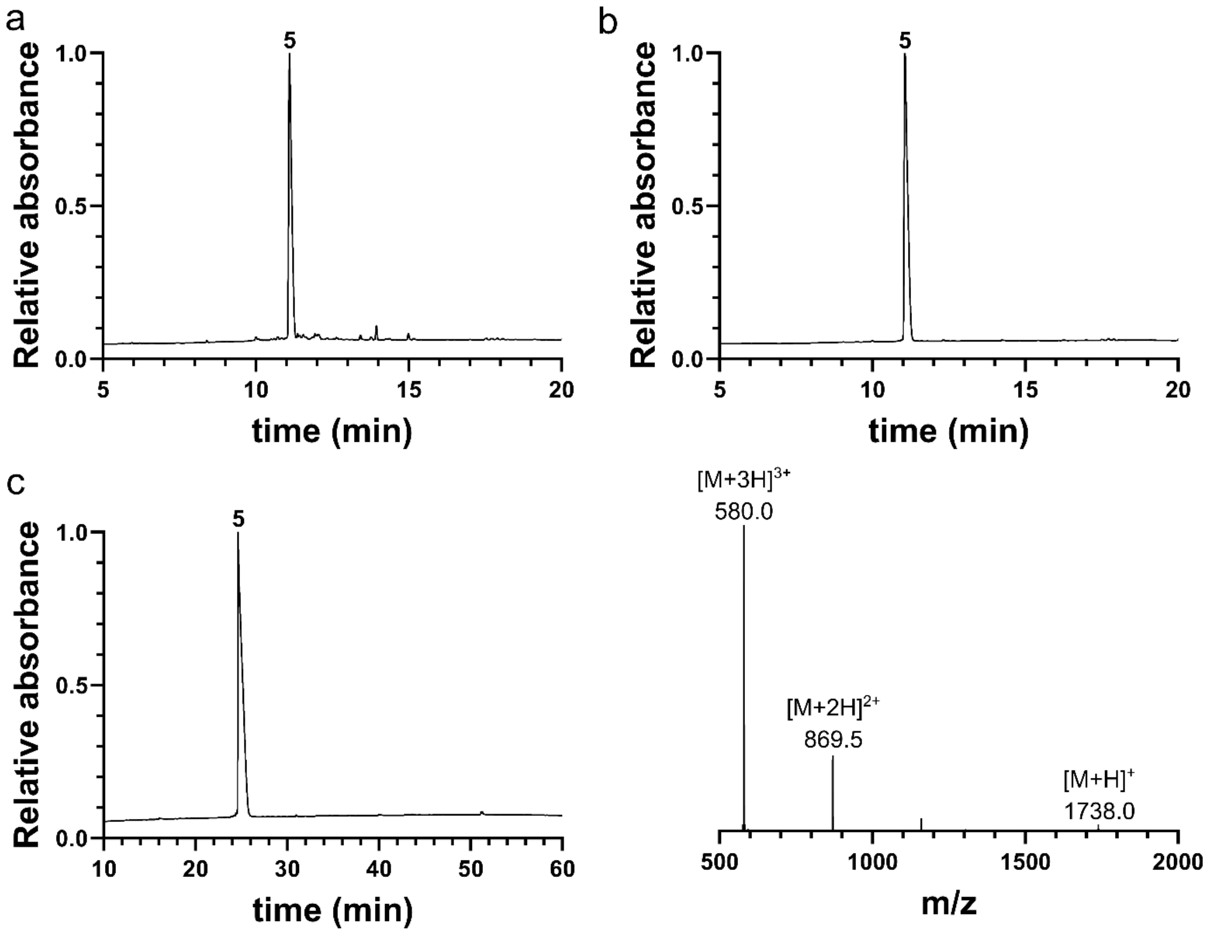

Supplementary Fig. 10: RP-HPLC traces and ESI-MS of NYAD-13 (**5**). **a**, Crude RP-HPLC trace (214 nm, 19 min 0–80% MeCN gradient). **b**, Purified RP-HPLC trace (214 nm, 19 min 0–80% MeCN gradient). **c**, Purified RP-HPLC trace (214 nm, 60 min 0–80% MeCN gradient) and ESI-MS.

5.6 Peptide–bismuth bicyclic complex (6)

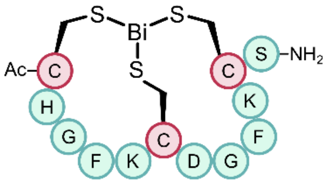

6

**Linear sequence:**	Ac-CHGFKCDGFKCS-NH_2 (12 aa)
**Resin:**	Fmoc-Rink Amide AM (0.42 mmol/g)
**Yield:**	64 mg (33%)
**Steps:**	29 chemical steps (627-unit operations)
**Synthesis time:**	26:23 (hh:mm)

The χDL file **6_Peptide–bismuth_bicyclic_complex.xdl** and the graph file **6_Peptide–bismuth_bicyclic_complex.json** were executed as described above. Blueprint output steps resulting from the χDL execution were as follows:

Step 1: *Add Fmoc-Rink Amide AM resin (238 mg) directly to spps_reactor.*
Step 2: Set stir rate of reactor_1 to 250 RPM.
Step 3: Resin_swell
Step 4: Coupling – **amino_acid** = Ser
Step 5: Coupling – **amino_acid** = Cys
Step 6: Coupling – **amino_acid** = Lys
Step 7: Coupling – **amino_acid** = Phe
Step 8: Coupling – **amino_acid** = Gly
Step 9: Coupling – **amino_acid** = Asp
Step 10: Coupling – **amino_acid** = Cys
Step 11: Coupling – **amino_acid** = Lys
Step 12: Coupling – **amino_acid** = Phe
Step 13: Coupling – **amino_acid** = Gly
Step 14: Coupling – **amino_acid** = His
Step 15: Coupling – **amino_acid** = Cys
Step 16: Acetylation – **capping_repeats** = 2
Step 17: Cleavage_and_workup – **peptide_solvent** = H_2O, **collection_flask** = reactor_1
Step 18: Reset liquid handling apparatus with H_2O (3 x 3 mL).
Step 19: Add H_2O (18.75 mL) directly to reactor_1 at default speed with stirring at 250 RPM.
Step 20: Add TRIS Buffer (62.5 mL) directly to reactor_1 at default speed with stirring at 250 RPM.
Step 21: Add TCEP (1.25 mL) directly to reactor_1 at default speed with stirring at 250 RPM.
Step 22: Add $BiBr_3$ (2.5 mL) directly to reactor_1 at 5 mL/min with stirring at 250 RPM.
Step 23*: Stir reactor_1 for 60 min at 250 RPM stopping stirring afterwards.
Step 24*: Reset liquid handling apparatus with DMF (3 x 3 mL).
Step 25: Shut down the platform.

The digital procedure was translated into the executed χDL file from literature reported methods[8].

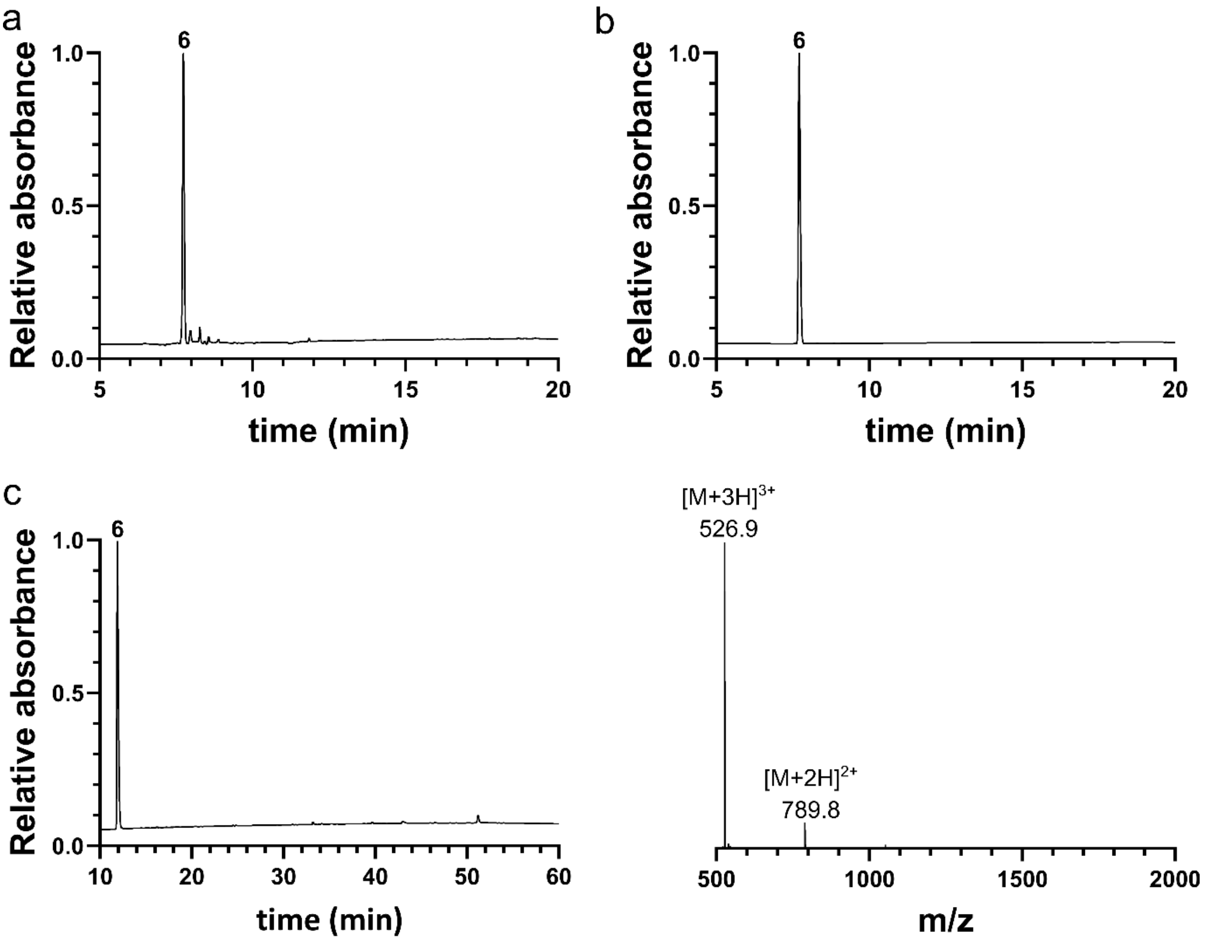

Supplementary Fig. 11: RP-HPLC traces and ESI-MS of peptide-bismuth bicyclic complex (**6**). **a**, Crude RP-HPLC trace (214 nm, 19 min 0–80% MeCN gradient). **b**, Purified RP-HPLC trace (214 nm, 19 min 0–80% MeCN gradient). **c**, Purified RP-HPLC trace (214 nm, 60 min 0–80% MeCN gradient) and ESI-MS.

5.7 CuAAC "click" 6-FAM fluorescent labelling of penetratin (7)

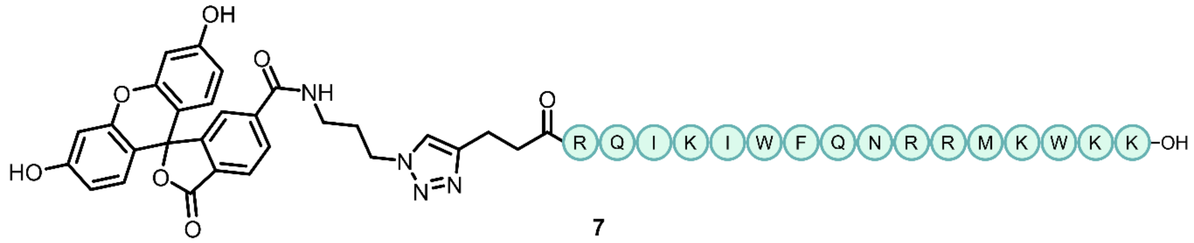

7

**Linear sequence:**	(Alkyne handle)RQIKIWFQNRRMKWKK-OH (16 aa, 1 alkyne handle)
**Resin:**	Fmoc-Lys(Boc)-Wang (0.50 mmol/g)
**Yield:**	52 mg (29%)
**Steps:**	35 chemical steps (817-unit operations)
**Synthesis time:**	42:53 (hh:mm)

The χDL file **7_CuAAC_click_6-FAM_fluorescent_labelling_of_penetratin.xdl** and the graph file **7_CuAAC_click_6-FAM_fluorescent_labelling_of_penetratin.json** were executed as described above. Blueprint output steps resulting from the χDL execution were as follows:

Step 1: *Add Fmoc-Lys(Boc)-Wang resin (100 mg) directly to spps_reactor.*
Step 2: *Add CuBr (323 mg) directly to reactor_1.*
Step 3: *Add 6-FAM-Azide (50 mg) directly to reactor_1.*
Step 4: Set stir rate of reactor_1 to 250 RPM.
Step 5: Resin_swell
Step 6: Coupling – **amino_acid** = Lys
Step 7: Coupling – **amino_acid** = Trp
Step 8: Coupling – **amino_acid** = Lys
Step 9: Coupling – **amino_acid** = Met
Step 10: Coupling – **amino_acid** = Arg
Step 11: Coupling – **amino_acid** = Arg
Step 12: Coupling – **amino_acid** = Asn
Step 13: Coupling – **amino_acid** = Gln
Step 14: Coupling – **amino_acid** = Phe
Step 15: Coupling – **amino_acid** = Trp
Step 16: Coupling – **amino_acid** = Ile
Step 17: Coupling – **amino_acid** = Lys
Step 18: Coupling – **amino_acid** = Ile
Step 19: Coupling – **amino_acid** = Gln
Step 20: Coupling – **amino_acid** = Arg
Step 21: Substrate_coupling – **substrate** = 4-pentynoic acid
Step 22: Deprotection
Step 23: Resin_wash
Step 24: Cleavage_workup_click – **peptide_solvent** = DMF, **collection_flask** = reactor_1
Step 25: Shut down the platform.

The digital procedure was translated into the executed χDL file from literature reported methods[9].

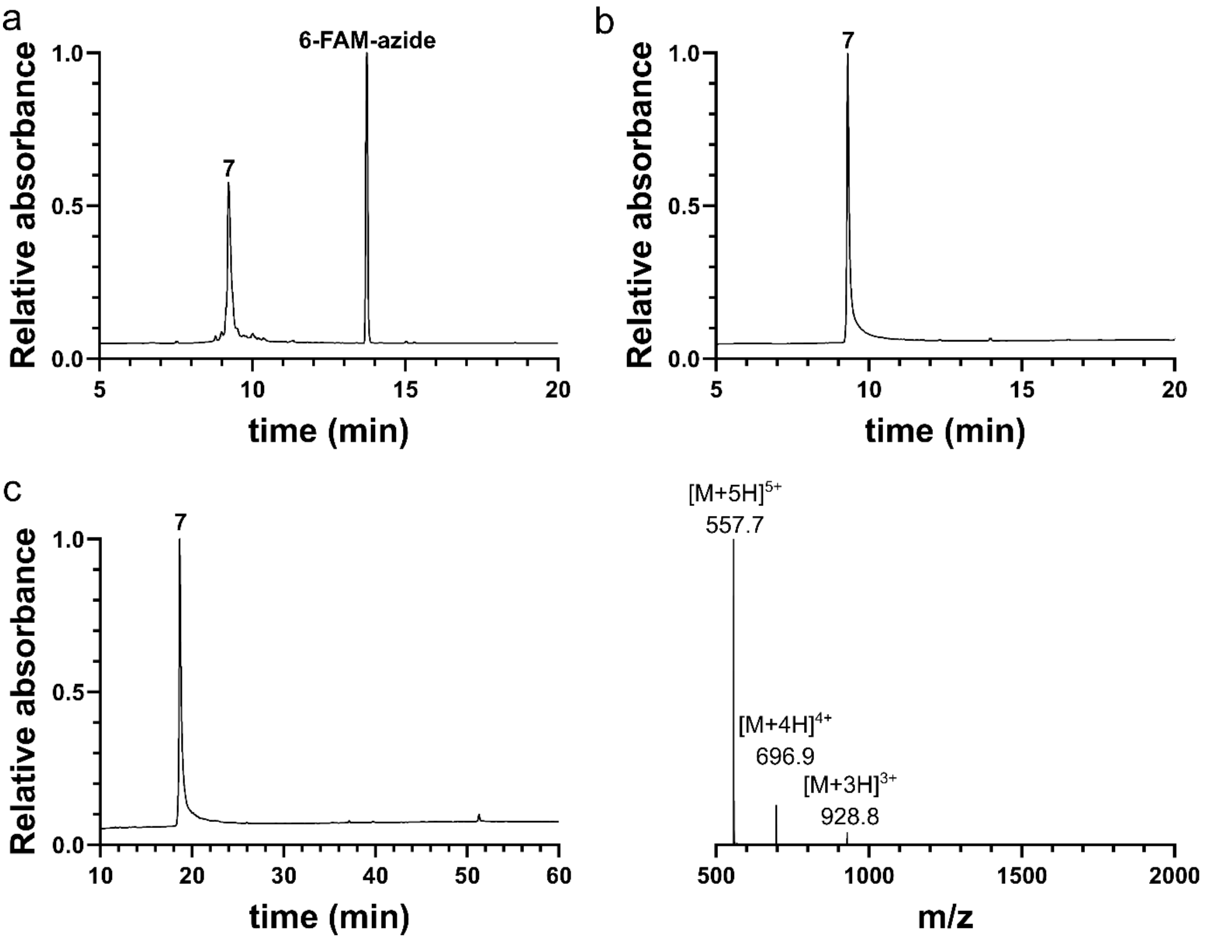

Supplementary Fig. 12: RP-HPLC traces and ESI-MS of CuAAC "click" 6-FAM fluorescent labelling of penetratin (**7**). **a**, Crude RP-HPLC trace (214 nm, 19 min 0–80% MeCN gradient). **b**, Purified RP-HPLC trace (214 nm, 19 min 0–80% MeCN gradient). **c**, Purified RP-HPLC trace (214 nm, 60 min 0–80% MeCN gradient) and ESI-MS.

5.8 Late-stage diversification via cysteine arylation stapling (8-11)

**Linear sequence:**	H-YCGGGCAL-OH (8 aa)	
**Resin:**	Fmoc-Leu-Wang resin (0.56 mmol/g)	
**Yield:**	**8**	6.0 mg (40%)
	**9**	8.2 mg (48%)
	**10**	6.6 mg (36%)
	**11**	5.4 mg (27%)
**Steps:**	18 chemical steps (417-unit operations)	
**Synthesis time:**	30:44 (hh:mm)	

The χDL file **8-11_Late-stage_diversification_via_cysteine_arylation_stapling.xdl** and the graph file **8-11_Late-stage_diversification_via_cysteine_arylation_stapling.json** were executed as described above. Blueprint output steps resulting from the χDL execution were as follows:

Step 1: *Add Fmoc-Leu-Wang resin (152 mg) directly to spps_reactor.*
Step 2: Set stir rate of reactor_1 to 250 RPM.
Step 3: Resin_swell
Step 4: Coupling – **amino_acid** = Ala
Step 5: Coupling – **amino_acid** = Cys
Step 6: Coupling – **amino_acid** = Gly
Step 7: Coupling – **amino_acid** = Gly
Step 8: Coupling – **amino_acid** = Gly
Step 9: Coupling – **amino_acid** = Cys
Step 10: Coupling – **amino_acid** = Tyr
Step 11: Deprotection
Step 12: Resin_wash
Step 13: Cleavage_and_workup – **peptide_solvent** = DMF, **collection_flask** = buffer_flask
Step 14: Peptide_stapling_diversification
Step 15: Shut down the platform.

The digital procedure was translated into the executed χDL file from literature reported methods[10].

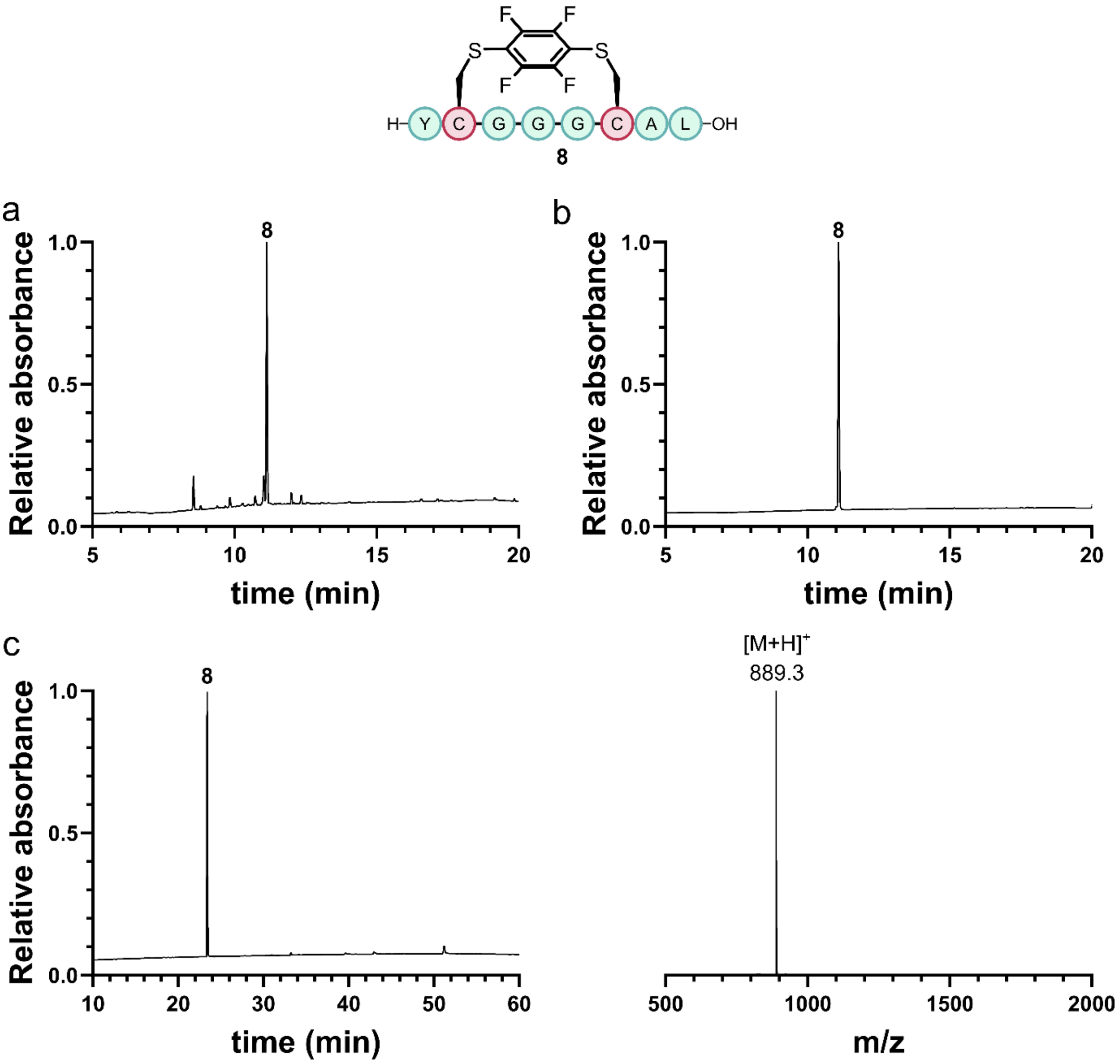

Supplementary Fig. 13: RP-HPLC traces and ESI-MS of late-stage diversification via cysteine arylation stapling (**8**). **a**, Crude RP-HPLC trace (214 nm, 19 min 0–80% MeCN gradient). **b**, Purified RP-HPLC trace (214 nm, 19 min 0–80% MeCN gradient). **c**, Purified RP-HPLC trace (214 nm, 60 min 0–80% MeCN gradient) and ESI-MS.

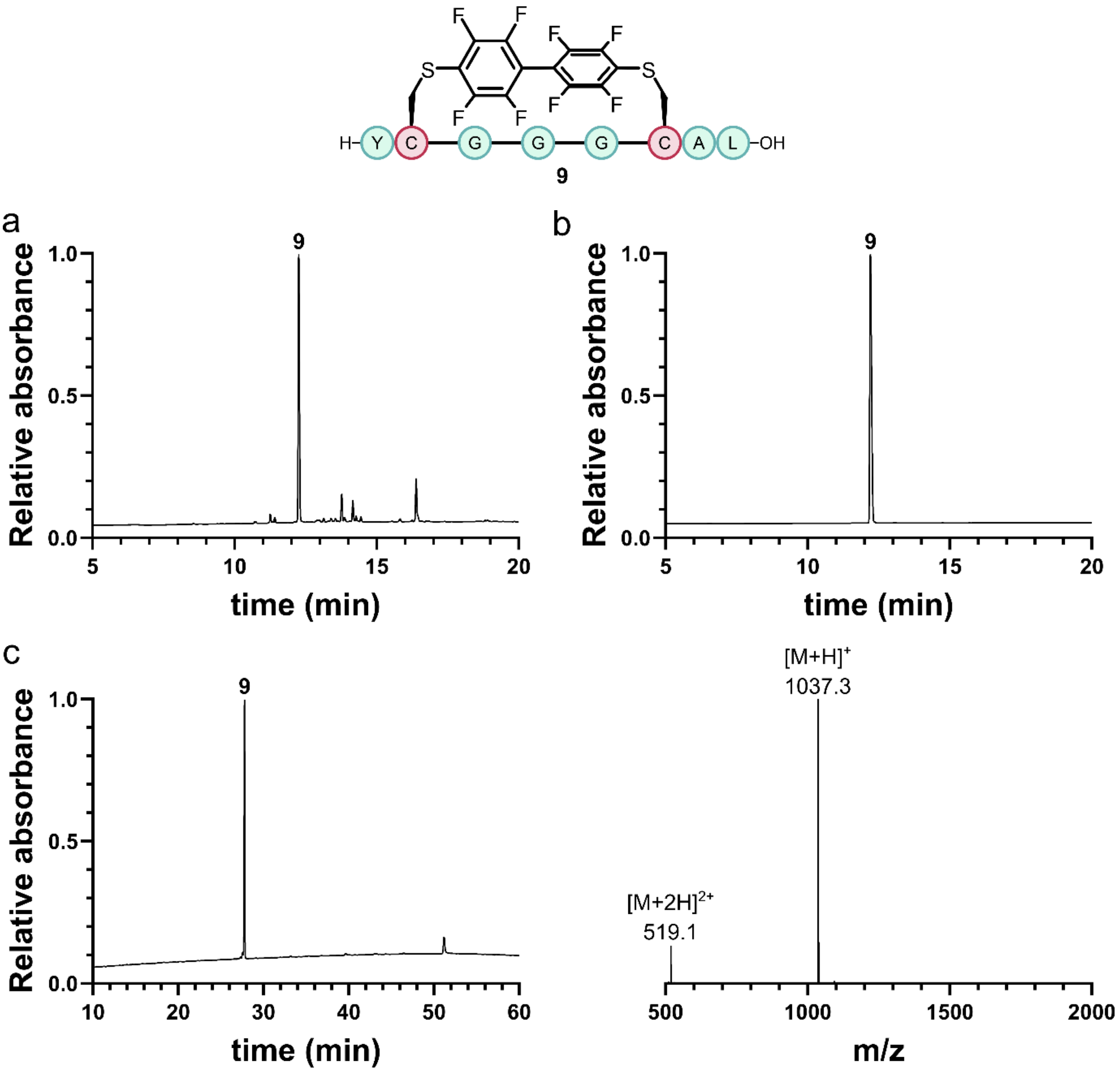

Supplementary Fig. 14: RP-HPLC traces and ESI-MS of late-stage diversification via cysteine arylation stapling (**9**). **a**, Crude RP-HPLC trace (214 nm, 19 min 0–80% MeCN gradient). **b**, Purified RP-HPLC trace (214 nm, 19 min 0–80% MeCN gradient). **c**, Purified RP-HPLC trace (214 nm, 60 min 0–80% MeCN gradient) and ESI-MS.

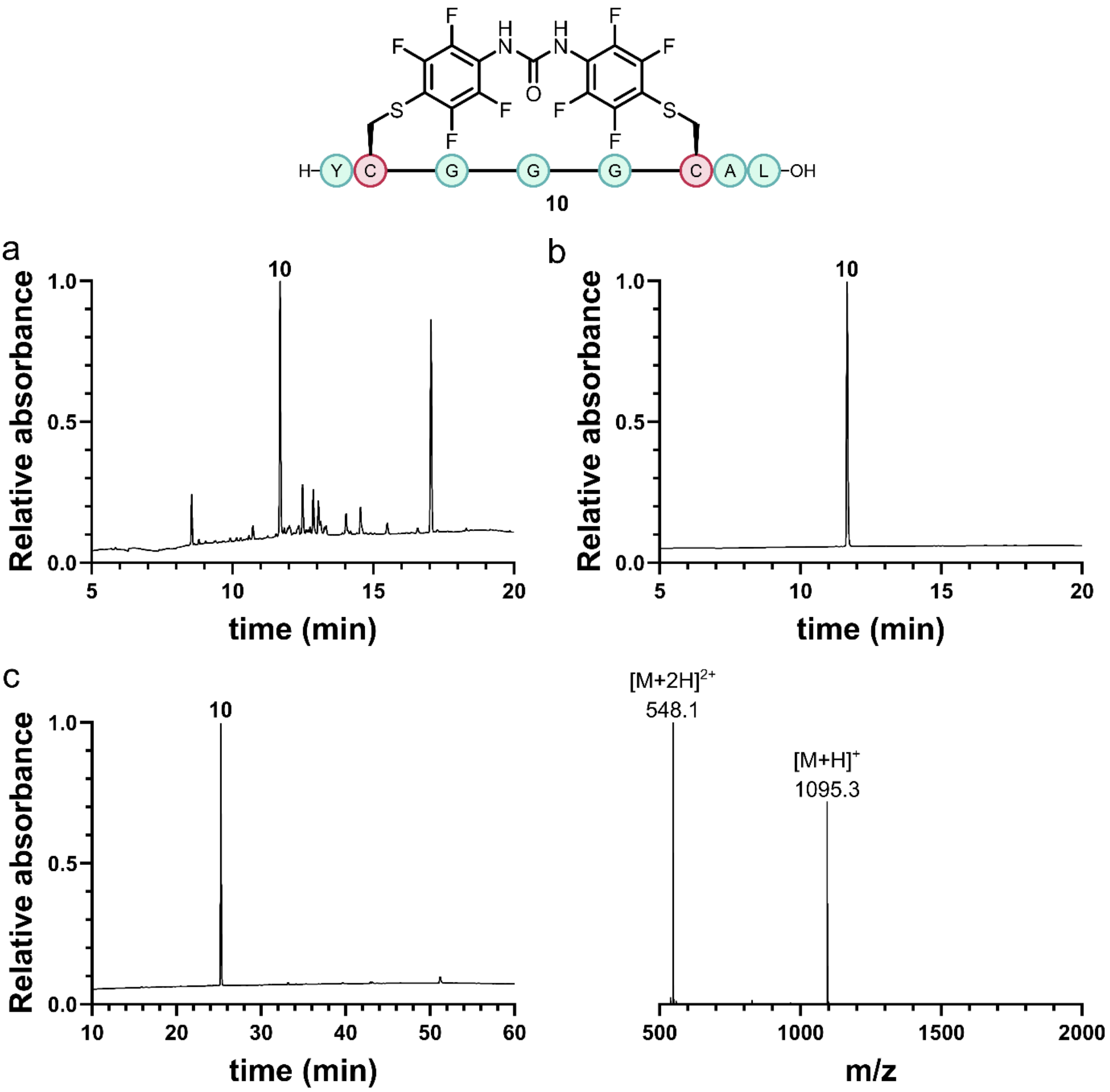

Supplementary Fig. 15: RP-HPLC traces and ESI-MS of late-stage diversification via cysteine arylation stapling (**10**). **a**, Crude RP-HPLC trace (214 nm, 19 min 0–80% MeCN gradient). **b**, Purified RP-HPLC trace (214 nm, 19 min 0–80% MeCN gradient). **c**, Purified RP-HPLC trace (214 nm, 60 min 0–80% MeCN gradient) and ESI-MS.

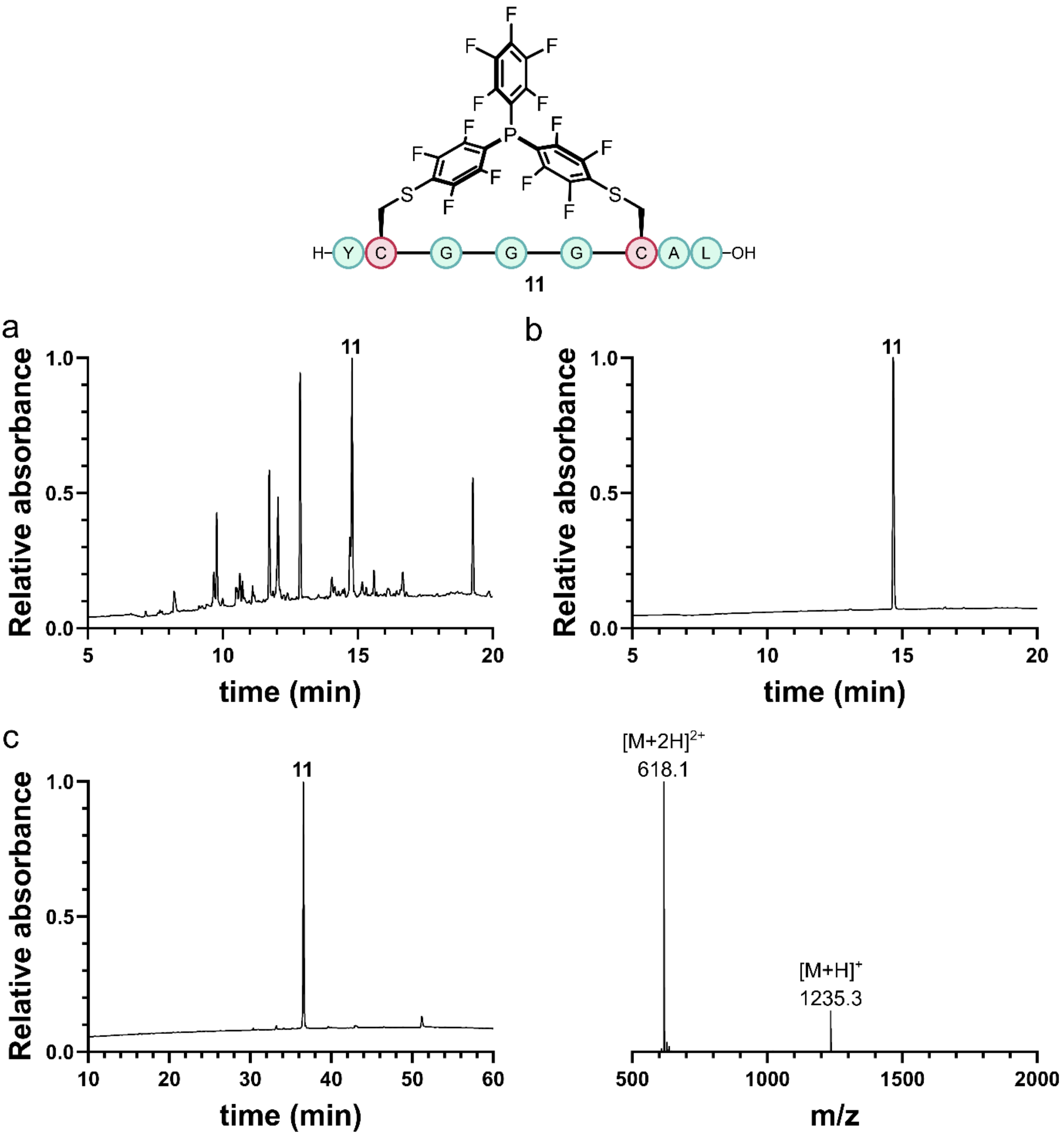

Supplementary Fig. 16: RP-HPLC traces and ESI-MS of late-stage diversification via cysteine arylation stapling (**11**). **a**, Crude RP-HPLC trace (214 nm, 19 min 0–80% MeCN gradient). **b**, Purified RP-HPLC trace (214 nm, 19 min 0–80% MeCN gradient). **c**, Purified RP-HPLC trace (214 nm, 60 min 0–80% MeCN gradient) and ESI-MS.

5.9 Capitellacin – directed oxidative folding (12)

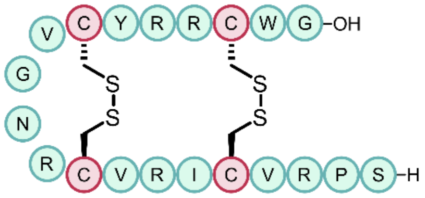

12c

**Linear sequence:**	H-SPRVC(Acm)IRVCRNGVCYRRC(Acm)WG-OH (20 aa)
**Resin:**	Fmoc-Gly-Wang (0.36 mmol/g)
**Yield:**	37 mg (24%)
**Steps:**	43 chemical steps (938-unit operations)
**Synthesis time:**	85:19 (hh:mm)

The χDL file **12_Capitellacin.xdl** and the graph file **12_Capitellacin.json** were executed as described above. Blueprint output steps resulting from the χDL execution were as follows:

Step 1: *Add Fmoc-Gly-Wang resin (139 mg) directly to spps_reactor.*
Step 2: Set stir rate of reactor_1 to 250 RPM.
Step 3: Reset liquid handling apparatus with H_2O (3 x 3 mL).
Step 4: Add ABC buffer (260 mL) directly to reactor_1 at default speed.
Step 5: Reset liquid handling apparatus with H_2O (3 x 3 mL).
Step 6: Resin_swell
Step 7: Coupling – **amino_acid** = Trp
Step 8: Coupling – **amino_acid** = Cys(Acm)
Step 9: Coupling – **amino_acid** = Arg
Step 10: Coupling – **amino_acid** = Arg
Step 11: Coupling – **amino_acid** = Tyr
Step 12: Coupling – **amino_acid** = Cys
Step 13: Coupling – **amino_acid** = Val
Step 14: Coupling – **amino_acid** = Gly
Step 15: Coupling – **amino_acid** = Asn
Step 16: Coupling – **amino_acid** = Arg
Step 17: Coupling – **amino_acid** = Cys
Step 18: Coupling – **amino_acid** = Val
Step 19: Coupling – **amino_acid** = Arg
Step 20: Coupling – **amino_acid** = Ile
Step 21: Coupling – **amino_acid** = Cys(Acm)
Step 22: Coupling – **amino_acid** = Val
Step 23: Coupling – **amino_acid** = Arg
Step 24: Coupling – **amino_acid** = Pro
Step 25: Coupling – **amino_acid** = Ser
Step 26: Deprotection
Step 27: Resin_wash
Step 28: Cleavage_and_workup – **peptide_solvent** = MeCN/H_2O (1:9, v/v), **collection_flask** = reactor_1
Step 29*: Stir reactor_1 for 48 h at 250 RPM stopping stirring afterwards.
Step 30*: Reset liquid handling apparatus with H_2O (3 x 3 mL).
Step 31: Add TFA (6 mL) directly to reactor_1 at default speed with stirring at 250 RPM.
Step 32: Add Iodine (2 mL) directly to reactor_1 at default speed with stirring at 250 RPM.

Step 33*: Stir reactor_1 for 30 min at 250 RPM stopping stirring afterwards.
Step 34*: Reset liquid handling apparatus with H_2O (3 x 3 mL).
Step 35: Add Ascorbic acid (6 mL) directly to reactor_1 at default speed with stirring at 250 RPM.
Step 36*: Stir reactor_1 for 30 min at 250 RPM stopping stirring afterwards.
Step 37*: Reset liquid handling apparatus with H_2O (3 x 3 mL).
Step 38: Shut down the platform.

The digital procedure was translated into the executed χDL file from literature reported methods[11,12].

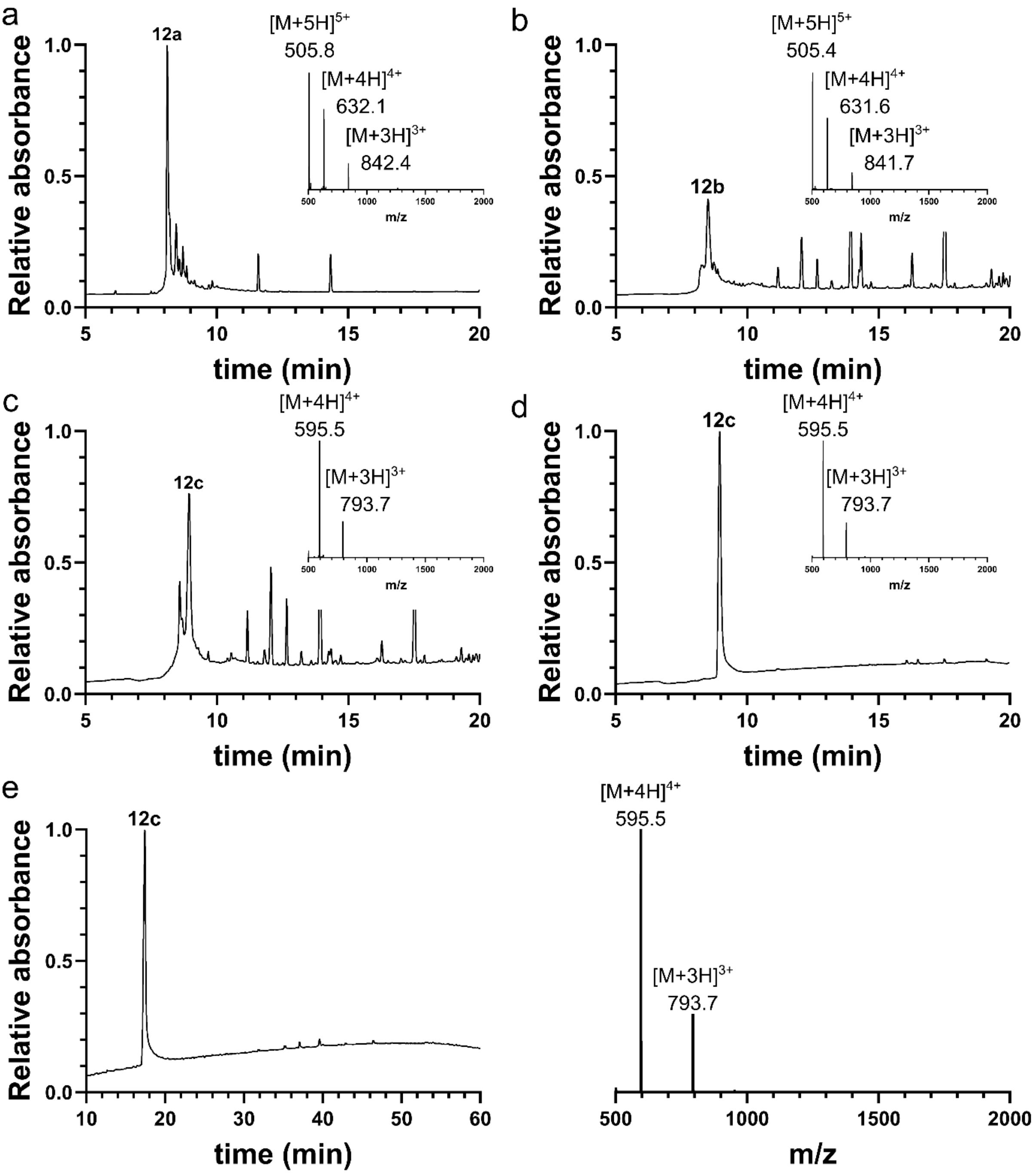

Supplementary Fig. 17: RP-HPLC traces and ESI-MS of Capitellacin. **a**, Crude RP-HPLC trace (214 nm, 19 min 0–80% MeCN gradient) and ESI-MS spectra (inlet) of Acm protected linear precursor (**12a**). **b**, Crude RP-HPLC trace (214 nm, 19 min 0–80% MeCN gradient) and ESI-MS spectra (inlet) of singly oxidized peptide (**12b**). **c**, Crude RP-HPLC trace (214 nm, 19 min 0–80% MeCN gradient) and ESI-MS spectra (inlet) of fully oxidized Capitellacin (**12c**). **d**, Purified RP-HPLC trace (214 nm, 19 min 0–80% MeCN gradient) and ESI-MS spectra (inlet) of fully oxidized Capitellacin (**12c**). **e**, RP-HPLC trace (214 nm, 60 min 0–80% MeCN gradient) and ESI-MS spectra of purified **12c**.

5.10 OPA-mediated sidechain cyclization and maleimide derivatization (13)

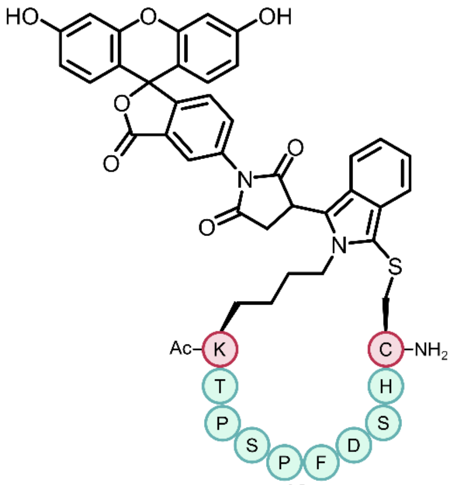

13c

Linear sequence: Ac-KTPSPFDSHC-NH_2 (10 aa)
Resin: Fmoc-Rink Amide AM (0.42 mmol/g)
Yield: 60 mg (67%)
Steps: 26 chemical steps (567-unit operations)
Synthesis time: 25:46 (hh:mm)

The χDL file **13_OPA-mediated_sidechain_cyclization_and_maleimide_derivatization.xdl** and the graph file **13_OPA-mediated_sidechain_cyclization_and_maleimide_derivatization.json** were executed as described above. Blueprint output steps resulting from the χDL execution were as follows:

Step 1: *Add Fmoc-Rink Amide AM resin (119 mg) directly to spps_reactor.*
Step 2: *Add OPA (50.5 mg) directly to flask_OPA.*
Step 3: *Add Mal-FAM (50 mg) directly to flask_Mal-FAM.*
Step 4: Set stir rate of reactor_1 to 250 RPM.
Step 5: Set stir rate of flask_OPA to 250 RPM.
Step 6: Set stir rate of flask_Mal-FAM to 250 RPM.
Step 7: Resin_swell
Step 8: Coupling – **amino_acid** = Cys
Step 9: Coupling – **amino_acid** = His
Step 10: Coupling – **amino_acid** = Ser
Step 11: Coupling – **amino_acid** = Asp
Step 12: Coupling – **amino_acid** = Phe
Step 13: Coupling – **amino_acid** = Pro
Step 14: Coupling – **amino_acid** = Ser
Step 15: Coupling – **amino_acid** = Pro
Step 16: Coupling – **amino_acid** = Thr
Step 17: Coupling – **amino_acid** = Lys
Step 18: Acetylation – **capping_repeats** = 2
Step 19: Cleavage_and_workup – **peptide_solvent** = PBS buffer, **collection_flask** = reactor_1
Step 20: OPA_cyclization
Step 21: Maleimide_derivatization
Step 22: Shut down the platform.

The digital procedure was translated into the executed χDL file from literature reported methods[13].

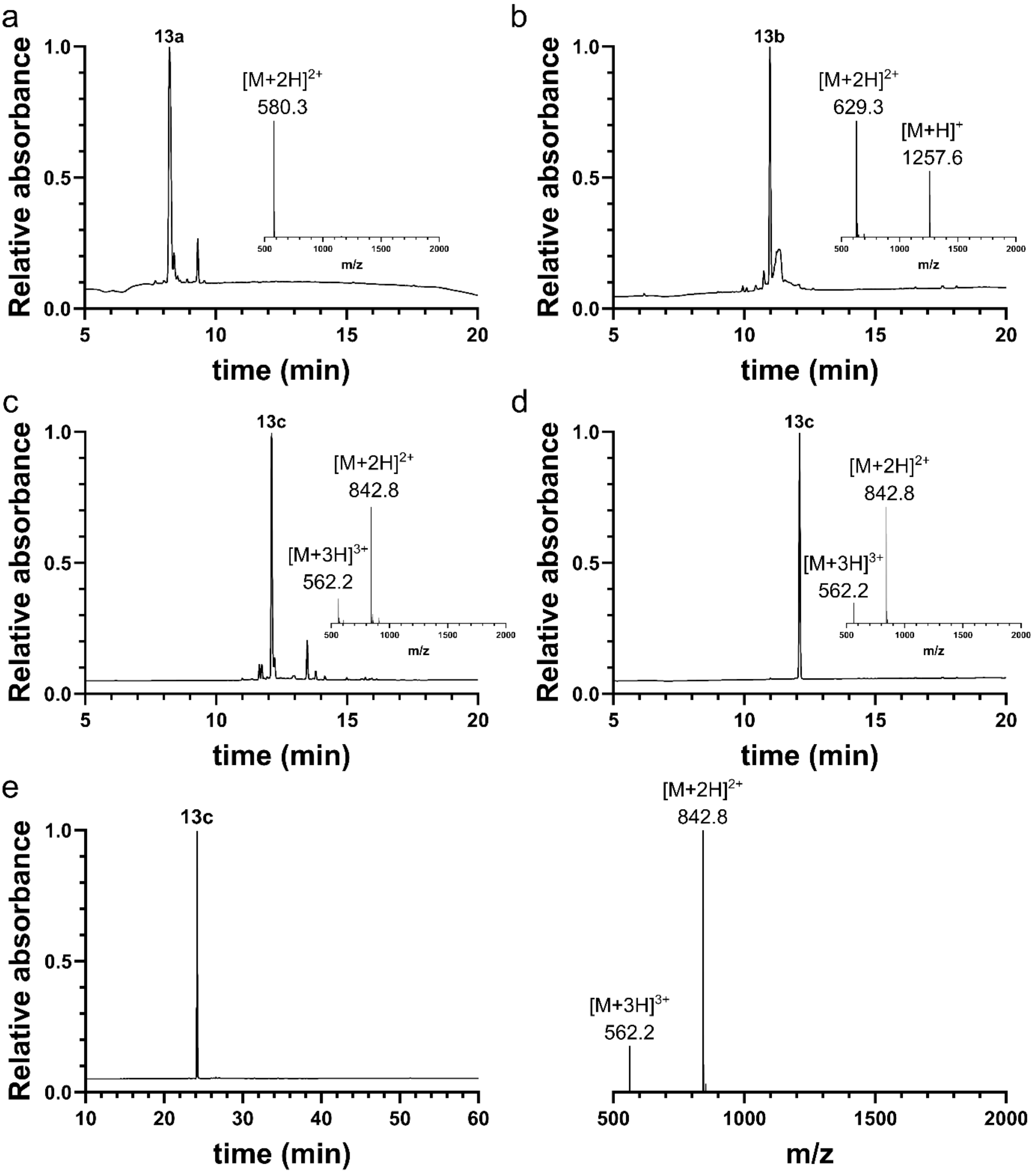

Supplementary Fig. 18: RP-HPLC traces and ESI-MS of OPA-mediated sidechain cyclization and maleimide derivatization reactions. **a**, Crude RP-HPLC trace (214 nm, 19 min 0–80% MeCN gradient) and ESI-MS spectra (inlet) of linear precursor (**13a**). **b**, Crude RP-HPLC trace (214 nm, 19 min 0–80% MeCN gradient) and ESI-MS spectra (inlet) of OPA-mediated sidechain cyclized peptide (**13b**). **c**, Crude RP-HPLC trace (214 nm, 19 min 0–80% MeCN gradient) and ESI-MS spectra (inlet) of cyclized and maleimide functionalized final product (**13c**). **d**, Purified RP-HPLC trace (214 nm, 19 min 0–80% MeCN gradient) and ESI-MS spectra (inlet) of cyclized and maleimide functionalized final product (**13c**). **e**, RP-HPLC trace (214 nm, 60 min 0–80% MeCN gradient) and ESI-MS spectra of purified **13c**.

5.11 α-amanitin analogue synthesis via native chemical ligation (14)

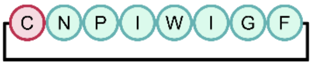

14c

Linear sequence: Ac-CNPIWIGF-$NHNH_2$ (8 aa)
Resin: 2-CTC resin (0.75 mmol/g)
Yield: 25 mg (36%)
Steps: 21 chemical steps (504-unit operations)
Synthesis time: 41:04 (hh:mm)

The χDL file **14_α-amanitin_analogue_synthesis_via_native_chemical_ligation.xdl** and the graph file **14_α-amanitin_analogue_synthesis_via_native_chemical_ligation.json** were executed as described above. Blueprint output steps resulting from the χDL execution were as follows:

Step 1: *Add 2-CTC resin (100 mg) directly to spps_reactor.*
Step 2: *Add MPAA (1.26 g) directly to reactor_1.*
Step 3: Set stir rate of reactor_1 to 250 RPM.
Step 4: Set stir rate of reactor_2 to 250 RPM.
Step 5: Resin_swell
Step 6: Resin_loading – **loading_reagent** = Fmoc-hydrazide, **loading_repeats** = 2, **loading_time** = 45 min
Step 7: Coupling – **amino_acid** = Phe
Step 8: Coupling – **amino_acid** = Gly
Step 9: Coupling – **amino_acid** = Ile
Step 10: Coupling – **amino_acid** = Trp
Step 11: Coupling – **amino_acid** = Ile
Step 12: Coupling – **amino_acid** = Pro
Step 13: Coupling – **amino_acid** = Asn
Step 14: Coupling – **amino_acid** = Cys
Step 15: Deprotection
Step 16: Resin_wash
Step 17: Cleavage_and_workup_for_NCL – **peptide_solvent** = Buffer A, **collection_flask** = reactor_1
Step 18: Thioesterification_and_NCL – **collection_flask** = reactor_1
Step 19: Shut down the platform.

The digital procedure was translated into the executed χDL file from literature reported methods[14,15].

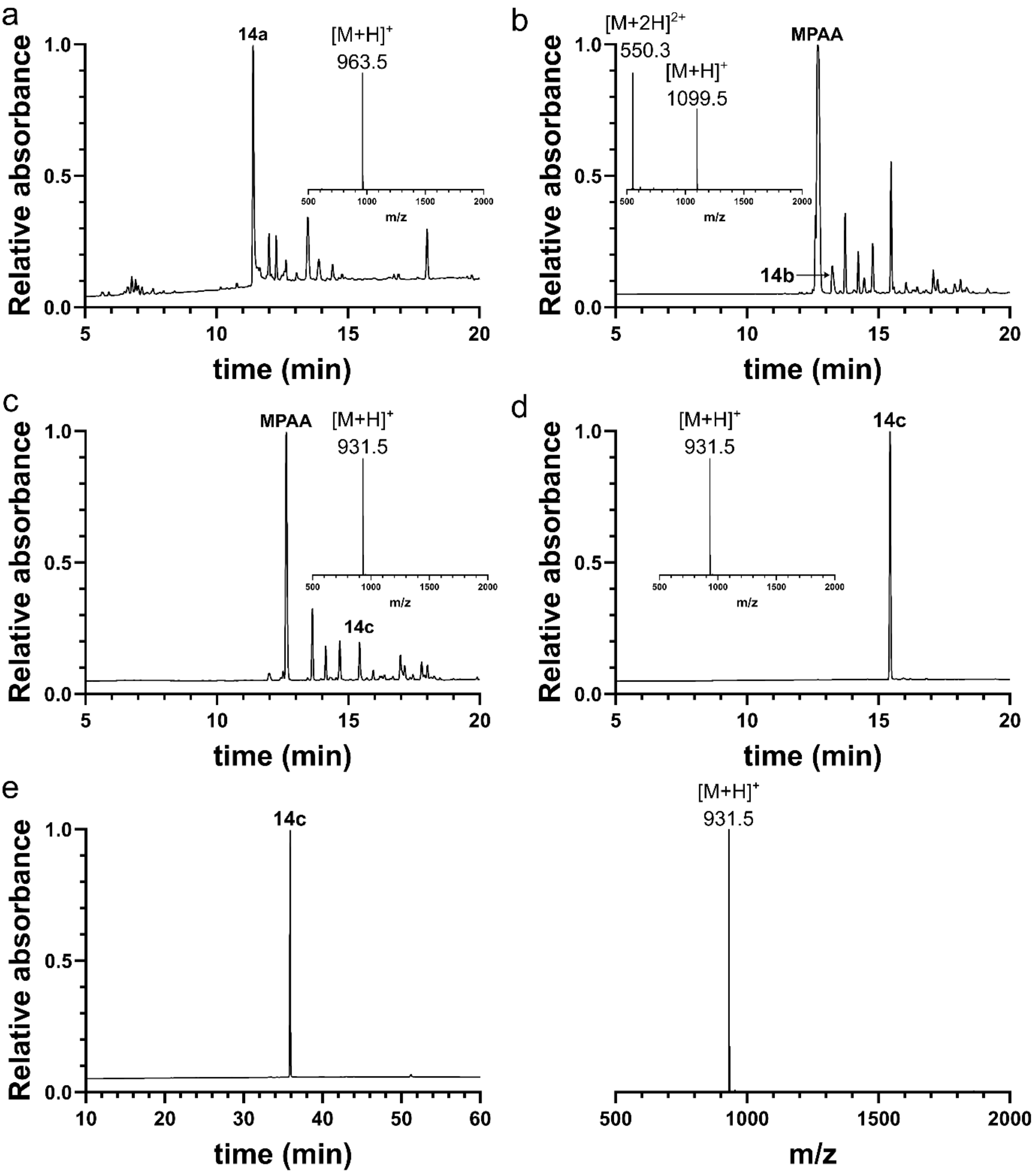

Supplementary Fig. 19: RP-HPLC traces and ESI-MS of α-amanitin analogue synthesis via native chemical ligation. **a**, Crude RP-HPLC trace (214 nm, 19 min 0–80% MeCN gradient) and ESI-MS spectra (inlet) of linear precursor (**14a**). **b**, Crude RP-HPLC trace (214 nm, 19 min 0–80% MeCN gradient) and ESI-MS spectra (inlet) of α-thioester (**14b**). **c**, Crude RP-HPLC trace (214 nm, 19 min 0–80% MeCN gradient) and ESI-MS spectra (inlet) of intramolecularly ligated final product (**14c**). **d**, Purified RP-HPLC trace (214 nm, 19 min 0–80% MeCN gradient) and ESI-MS intramolecularly ligated final product (**14c**). **e**, RP-HPLC trace (214 nm, 60 min 0–80% MeCN gradient) and ESI-MS spectra of purified **14c**.

5.12 N-Methyl-18A (15)

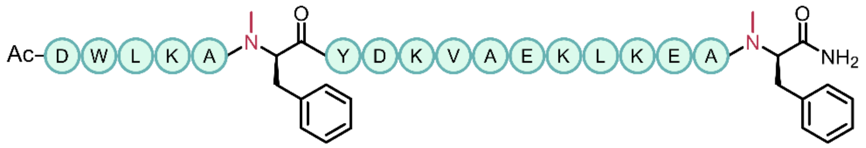

15

Linear sequence: Ac-DWLKA(N-Me-F)YDKVAEKLKEA(N-Me-F)-NH_2 (18 aa) (**15**)
Resin: Fmoc-Rink Amide AM (0.42 mmol/g)
Crude purity: 79% by RP-HPLC at 214 nm
Crude yield: 213 mg (62%)
Steps: 40 chemical steps, 916-unit operations
Synthesis time: 34:21 (hh:mm)

The χDL file **15_N-Methyl-18A.xdl** and the graph file **15_N-Methyl-18A.json** were executed as described above. Blueprint output steps resulting from the χDL execution were as follows:

Step 1: *Add Fmoc-Rink Amide AM resin (238 mg) directly to spps_reactor.*
Step 2: Resin_swell
Step 3: Coupling – **amino_acid** = N-Me-Phe
Step 4: Coupling – **amino_acid** = Ala
Step 5: Coupling – **amino_acid** = Glu
Step 6: Coupling – **amino_acid** = Lys
Step 7: Coupling – **amino_acid** = Leu
Step 8: Coupling – **amino_acid** = Lys
Step 9: Coupling – **amino_acid** = Glu
Step 10: Coupling – **amino_acid** = Ala
Step 11: Coupling – **amino_acid** = Val
Step 12: Coupling – **amino_acid** = Lys
Step 13: Coupling – **amino_acid** = Asp
Step 14: Coupling – **amino_acid** = Tyr
Step 15: Coupling – **amino_acid** = N-Me-Phe
Step 16: Coupling – **amino_acid** = Ala
Step 17: Coupling – **amino_acid** = Lys
Step 18: Coupling – **amino_acid** = Leu
Step 19: Coupling – **amino_acid** = Trp
Step 20: Coupling – **amino_acid** = Asp
Step 21: Acetylation – **capping_repeats** = 2
Step 22: Cleavage_and_workup – **peptide_solvent** = MeCN/H_2O (50:50 v/v), **collection_flask** = product_flask
Step 23: Shut down the platform.

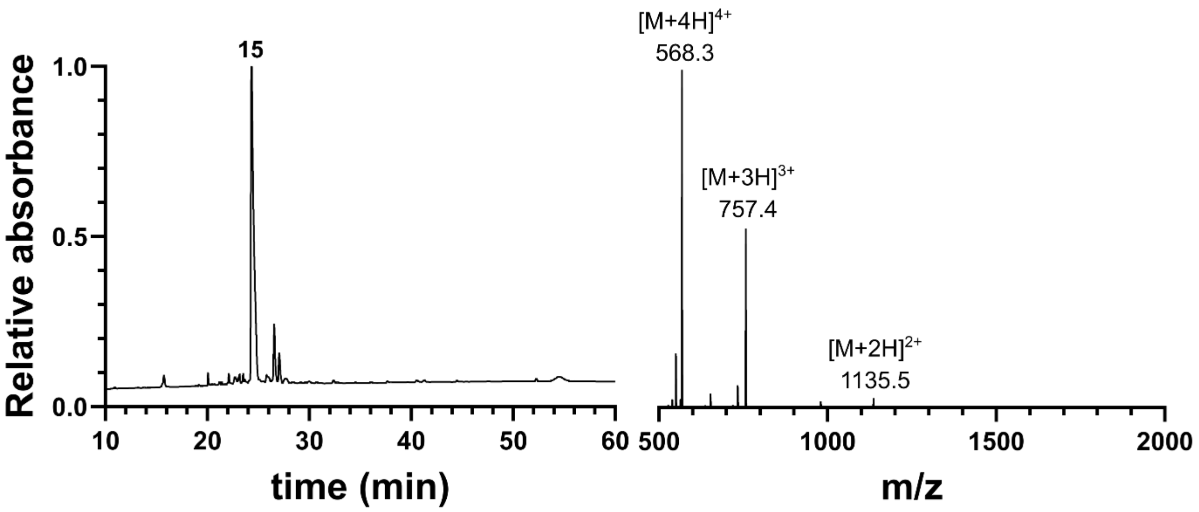

Supplementary Fig. 20: Crude RP-HPLC (214 nm) and ESI-MS analysis of N-Methyl-18A (**15**).

6. Supplementary References

1. Rohrbach, S. *et al.* Digitization and validation of a chemical synthesis literature database in the ChemPU. *Science* **377**, 172–180 (2022).
2. Leonov, A. I. *et al.* An integrated self-optimizing programmable chemical synthesis and reaction engine. *Nat. Commun.* **15**, 1240 (2024).
3. Steiner, S. *et al.* Organic synthesis in a modular robotic system driven by a chemical programming language. *Science* **363**, eaav2211 (2019).
4. Angelone, D. *et al.* Convergence of multiple synthetic paradigms in a universally programmable chemical synthesis machine. *Nat. Chem.* **13**, 63–69 (2021).
5. Lau, J. *et al.* Discovery of the Once-Weekly Glucagon-Like Peptide-1 (GLP-1) Analogue Semaglutide. *J. Med. Chem.* **58**, 7370–7380 (2015).
6. Østergaard, S. *et al.* The effect of fatty diacid acylation of human PYY3-36 on Y2 receptor potency and half-life in minipigs. *Sci. Rep.* **11**, 21179 (2021).
7. Zhang, H. *et al.* A Cell-penetrating Helical Peptide as a Potential HIV-1 Inhibitor. *J. Mol. Biol.* **378**, 565–580 (2008).
8. Voss, S., Rademann, J. & Nitsche, C. Peptide–Bismuth Bicycles: In Situ Access to Stable Constrained Peptides with Superior Bioactivity. *Angew. Chem. Int. Ed.* **61**, e202113857 (2022).
9. Wolfe, J. M. *et al.* Machine Learning To Predict Cell-Penetrating Peptides for Antisense Delivery. *ACS Cent. Sci.* **4**, 512–520 (2018).
10. Spokoyny, A. M. *et al.* A Perfluoroaryl-Cysteine SNAr Chemistry Approach to Unprotected Peptide Stapling. *J. Am. Chem. Soc.* **135**, 5946–5949 (2013).
11. Jin, A.-H. *et al.* Molecular Engineering of Conotoxins: The Importance of Loop Size to α-Conotoxin Structure and Function. *J. Med. Chem.* **51**, 5575–5584 (2008).
12. Rehm, F. B. H. *et al.* Repurposing a plant peptide cyclase for targeted lysine acylation. *Nat. Chem.* **16**, 1481–1489 (2024).

13. Zhang, Y., Zhang, Q., Wong, C. T. T. & Li, X. Chemoselective Peptide Cyclization and Bicyclization Directly on Unprotected Peptides. *J. Am. Chem. Soc.* **141**, 12274–12279 (2019).

14. Bird, M. J. & Dawson, P. E. A shelf stable Fmoc hydrazine resin for the synthesis of peptide hydrazides. *Pept. Sci.* **114**, e24268 (2022).

15. Cistrone, P. A. *et al.* Native Chemical Ligation of Peptides and Proteins. *Curr. Protoc. Chem. Biol.* **11**, e61 (2019).
